# HyGAnno: hybrid graph neural network–based cell type annotation for single-cell ATAC sequencing data

**DOI:** 10.1093/bib/bbae152

**Published:** 2024-04-05

**Authors:** Weihang Zhang, Yang Cui, Bowen Liu, Martin Loza, Sung-Joon Park, Kenta Nakai

**Affiliations:** Department of Computational Biology and Medical Sciences, Graduate school of Frontier Sciences, University of Tokyo, Tokyo, Japan; Department of Computational Biology and Medical Sciences, Graduate school of Frontier Sciences, University of Tokyo, Tokyo, Japan; Department of Computational Biology and Medical Sciences, Graduate school of Frontier Sciences, University of Tokyo, Tokyo, Japan; Human Genome Center, Institute of Medical Science, University of Tokyo, Tokyo, Japan; Human Genome Center, Institute of Medical Science, University of Tokyo, Tokyo, Japan; Department of Computational Biology and Medical Sciences, Graduate school of Frontier Sciences, University of Tokyo, Tokyo, Japan; Human Genome Center, Institute of Medical Science, University of Tokyo, Tokyo, Japan

**Keywords:** single-cell ATAC sequencing, label transfer learning, graph embedding, graph neural network

## Abstract

Reliable cell type annotations are crucial for investigating cellular heterogeneity in single-cell omics data. Although various computational approaches have been proposed for single-cell RNA sequencing (scRNA-seq) annotation, high-quality cell labels are still lacking in single-cell sequencing assay for transposase-accessible chromatin (scATAC-seq) data, because of extreme sparsity and inconsistent chromatin accessibility between datasets. Here, we present a novel automated cell annotation method that transfers cell type information from a well-labeled scRNA-seq reference to an unlabeled scATAC-seq target, via a parallel graph neural network, in a semi-supervised manner. Unlike existing methods that utilize only gene expression or gene activity features, HyGAnno leverages genome-wide accessibility peak features to facilitate the training process. In addition, HyGAnno reconstructs a reference–target cell graph to detect cells with low prediction reliability, according to their specific graph connectivity patterns. HyGAnno was assessed across various datasets, showcasing its strengths in precise cell annotation, generating interpretable cell embeddings, robustness to noisy reference data and adaptability to tumor tissues.

## Graphical Abstract

**Graphical Abstract:**
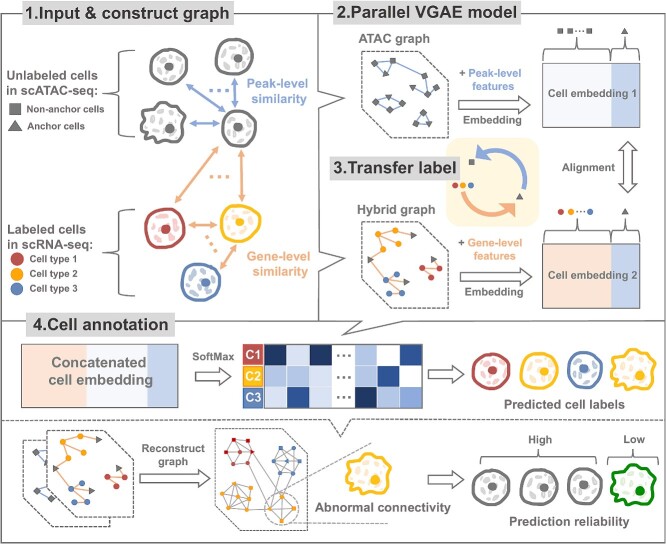

## INTRODUCTION

Single-cell sequencing technologies have become increasingly popular in contemporary biology and promoted the understanding of the molecular mechanisms underlying the formation of heterogeneous cell populations [1–3]. In particular, multi-omics approaches coupled with chromatin accessibility using single-cell ATAC sequencing (scATAC-seq) and transcriptome analysis using single-cell RNA sequencing (scRNA-seq) are widely used to dissect gene regulation at the single-cell level. While numerous studies have achieved computational and experimental improvements by jointly analyzing scRNA-seq and scATAC-seq data [4, 5], the prerequisite is that cell populations in two modalities must be annotated and invariably matched, which imposes a high demand on designing effective cell type annotation tools.

Cell type annotation problem is initially considered in the context of scRNA-seq [6–8] and can be majorly solved in two ways [9, 10]: (1) manual labeling: after cell clustering with unsupervised approaches, biologists manually annotate each cell cluster by comparing its differential expression genes with well-known gene markers. When countless cell clusters are detected, such approaches are not only time-consuming but also in low accuracy; (2) Automatic labeling: transferring cell labels from well-labeled scRNA-seq reference to unlabeled scRNA-seq target based on computational approaches [7, 11]. As both reference and target data are from the same modality, the performance of automatic labeling methods is thus ensured. When excessive attention is concentrated on the cell type annotation problem of the scRNA-seq data, the same issue for scATAC-seq data is being seriously neglected. Completely distinct from gene features in scRNA-seq, the feature set of scATAC-seq involves the accessible peaks (open chromatin regions) enriched by aligned reads [12], thus being distinct among different datasets. Even worse, peaks containing innumerable *cis-*regulatory elements (CREs) such as promoters, enhancers and transcription factor binding sites cause extremely high dimensional count matrix. Meanwhile, due to low copy numbers of DNA, only a maximum of 10% peaks can be finally detected in each cell; the data sparsity of scATAC-seq is thereby inherent and unavoidable [13].

To overcome the paucity of cell type annotation methods for scATAC-seq, many studies have employed the following three strategies, with room for improvement: (1) mapping peaks to important genes. Cells with peaks enriched in gene signatures or gene markers are manually annotated by experts. However, owing to the high sparsity of peaks and immature establishment of libraries, cell type annotation based on this strategy is of low resolution; (2) incorporating the cell labels of scRNA-seq data as references [14–18], such as Seurat v3 [15], scJoint [16], scGCN [17] and Conos [18]. As one necessary preprocessing of these methods, the genome-wide accessible peak (peak-level) features of the target scATAC-seq must be transformed into gene activity (gene-level) features [19, 20]. Then, Seurat v3, Conos and scJoint adopt conventional cell type annotation strategies designed for scRNA-seq, which transfer cell labels from reference cells to target cells according to their gene-level similarity. Although these tools have been widely used because of the ease of obtaining high-quality scRNA-seq references, the inherent peak-level information is not being used to its full potential, leading impossibility of these methods in detecting cell type–specific peaks. Applying an advanced graph convolution network (GCN) [21], scGCN successfully embeds the target cell graph and the reference–target cell graph in one shared latent space, where labeled and unlabeled cells are clustered together if they have similar graph structures. Whereas the most informative reference cell graph is not used in the training process of scGCN, potentially limiting its efficiency; (3) incorporating the cell labels of scATAC-seq data as references, such as Cellcano [22] and EpiAnno [23]. Cellcano converts both the reference and target scATAC-seq data into gene activity matrices, resulting in the same peak-level information loss problem. Although EpiAnno trains a model with peak-level features by a Bayesian network, the availability of well-annotated scATAC-seq datasets that can serve as references is limited, potentially constraining its efficiency. Therefore, there is an imperative need for methods that can be harmonious in blending both gene-level features and peak-level features in the training process, meanwhile using high-quality scRNA-seq reference for high-resolution annotation.

To solve the limitations mentioned above, in this study, we proposed a novel graph-based deep learning approach called HyGAnno to predict cell types in scATAC-seq experiments. Our method represents the cell populations in scRNA-seq and scATAC-seq datasets as undirected graphs and links the graphs using anchor cells. These graphs are then embedded and learned using graph neural networks, to transfer the cell labels of the scRNA-seq data to the corresponding cell populations in the scATAC-seq data. The main contribution of HyGAnno is that it trains the model with both peak-level features and gene-level features based on graph concept, ensuring precise cell annotations and offering a potential way for the detection of cell type–specific peaks. Besides, the target–reference cell graph generated by the graph decoder part of the HyGAnno can be used to further describe the prediction reliability.

## MATERIAL AND METHODS

### Graph construction and anchor cell detection

After applying Seurat [15] and Signac [20] for quality control, a gene expression matrix ${\mathbf{X}}^{\boldsymbol{rna}}\in{\mathbb{R}}^{g\times{n}_1}$ from scRNA-seq data and a peak matrix ${\mathbf{X}}^{\boldsymbol{atac}}\in{\mathbb{R}}^{p\times{n}_2}$ from scATAC-seq data are generated, where $g$ is the number of highly variable genes, *p* is the number of top accessible peaks and ${n}_1$ and ${n}_2$ are the cell numbers. Meanwhile, ${\mathbf{X}}^{\boldsymbol{atac}}$ is transferred to a gene activity matrix ${\mathbf{X}}^{\boldsymbol{gam}}\in{\mathbb{R}}^{g\times{n}_2}$ by calculating the enrichment of accessible peaks in highly variable genes [19, 20]. We first employed principal component analysis (PCA) on ${\mathbf{X}}^{\boldsymbol{rna}}$ and latent semantic indexing (LSI) on ${\mathbf{X}}^{\boldsymbol{atac}}$, projecting them into two separated $n$-dimensional spaces, respectively. Then, RNA cell graph ${\mathbf{G}}^{\boldsymbol{rna}}\in{\mathbb{R}}^{n_1\times{n}_1}$ and ATAC cell graph ${\mathbf{G}}^{\boldsymbol{atac}}\in{\mathbb{R}}^{n_2\times{n}_2}$ are constructed by utilizing shared $k$-nearest neighbors (${k}_1$) within these low-dimensional spaces. Next, we leveraged canonical correlation analysis (CCA) [24] to project the standardized ${\mathbf{X}}^{\boldsymbol{rna}}$ and ${\mathbf{X}}^{\boldsymbol{gam}}$ into a shared $n$-dimensional space [17], where two cells from different modalities are defined as a pair of anchor cells if they are in the shared *k*-nearest neighbors $({k}_2)$ of each other. After that, all anchor cells are added to ${\mathbf{G}}^{\boldsymbol{rna}}$, yielding an extend hybrid graph ${\mathbf{G}}^{\boldsymbol{H}}\in{\mathbb{R}}^{\left({n}_1+\left|\mathbb{A}\right|\right)\times \left({n}_1+\left|\mathbb{A}\right|\right)}$ and its feature matrix ${\mathbf{X}}^{\boldsymbol{H}}=\Big[{\overset{\sim }{\mathbf{X}}}^{\boldsymbol{rna}},{\overset{\sim }{\mathbf{X}}}_{\mathbb{A}}^{\boldsymbol{gam}}\Big]\in{\mathbb{R}}^{g\times \left({\text{n}}_1+\left|\mathbb{A}\right|\right)}$, where $\left|\ast \right|$ represents the size of a set; $\mathbb{A}$ is the set of ATAC anchor cells; and ${\overset{\sim }{\mathbf{X}}}^{\boldsymbol{rna}}\in{\mathbb{R}}^{g\times{n}_1}$ and ${\overset{\sim }{\mathbf{X}}}_{\mathbb{A}}^{\boldsymbol{gam}}\in{\mathbb{R}}^{g\times \left|\mathbb{A}\right|}$ are the standardized feature matrices of ${\mathbf{X}}^{\boldsymbol{rna}}$ and ${\mathbf{X}}^{\boldsymbol{gam}}$, respectively. Finally, we have the graph set $\mathbb{G}=\left\{{\mathbf{G}}^{\boldsymbol{H}},{\mathbf{G}}^{\boldsymbol{atac}}\right\}$ and feature set $\mathbb{X}=\Big\{{\mathbf{X}}^{\boldsymbol{H}},{\overset{\sim }{\mathbf{X}}}^{\boldsymbol{atac}}\Big\}$ for the following graph neural networks, where ${\overset{\sim }{\mathbf{X}}}^{\boldsymbol{atac}}$ is the term frequency-inverse document frequency (TF-IDF) normalized ${\mathbf{X}}^{\boldsymbol{atac}}$. More details of the graph construction and selection of anchor cell numbers are provided in Supplementary Notes 1 and 2, respectively. The properties of the constructed graphs are recorded in Supplementary Table 1.

### Parallel variational graph auto-encoders

To transfer the cell labels from the hybrid graph to the ATAC graph, a parallel variational graph auto-encoder (VGAE) [25] framework embeds graphs in $\mathbb{G}$ together with $\mathbb{X}$ into $k$*-*dimensional spaces, as follows:


(1) \begin{equation*} {\mathbf{Z}}^{\boldsymbol{H}}\in{\mathbb{R}}^{k\times \left({n}_1+\left|\mathbb{A}\right|\right)}={\mathbf{N}}^{\boldsymbol{H}}\odot{\mathbf{Z}}_{\boldsymbol{\sigma}}^{\boldsymbol{H}}+{\mathbf{Z}}_{\boldsymbol{\mu}}^{\boldsymbol{H}}, \end{equation*}

(2) \begin{equation*} {\displaystyle \begin{array}{c}{\mathbf{Z}}^{\boldsymbol{atac}}\in{\mathbb{R}}^{k\times{n}_2}={\mathbf{N}}^{\boldsymbol{atac}}\odot{\mathbf{Z}}_{\boldsymbol{\sigma}}^{\boldsymbol{atac}}+{\mathbf{Z}}_{\boldsymbol{\mu}}^{\boldsymbol{atac}},\end{array}} \end{equation*}

where ${\mathbf{N}}^{\boldsymbol{H}}\in{\mathbb{R}}^{k\times \left({n}_1+\left|\mathbb{A}\right|\right)}$ and ${\mathbf{N}}^{\boldsymbol{atac}}\in{\mathbb{R}}^{k\times{n}_2}$ are random standard normal matrices; $\mathbf{A}\odot \mathbf{B}$ represents the Hadamard product of two matrices; and ${\mathbf{Z}}_{\boldsymbol{\mu}}^{\boldsymbol{H}},{\mathbf{Z}}_{\boldsymbol{\mu}}^{\boldsymbol{atac}},{\mathbf{Z}}_{\boldsymbol{\sigma}}^{\boldsymbol{H}}$and ${\mathbf{Z}}_{\boldsymbol{\sigma}}^{\boldsymbol{atac}}$ are calculated using a two-layer GCN [21] with dimension number $d$ of the hidden layer and the dimension number $k$ of the output layer (Supplementary Note 3). Finally, the loss function regulating the latent variables ${\mathbf{Z}}^{\boldsymbol{H}}$and ${\mathbf{Z}}^{\boldsymbol{atac}}$ in the two VGAEs is optimized as follows:


(3) \begin{equation*} {\displaystyle \begin{array}{c}{L}_1=-\frac{1}{2}{\sum}_{d=1}^k\left({\sum}_{i=1}^{n_1+\left|\mathbb{A}\right|}{f}_{d,i}^H+{\sum}_{j=1}^{n_2}{f}_{d,j}^{atac}\right),\end{array}} \end{equation*}

(4) \begin{equation*} {\displaystyle \begin{array}{c}{f}_{d,i}^H=1+2\mathit{\log}\left({\sigma}_{d,i}^H\right)-{\left({\mu}_{d,i}^H\right)}^2-{\left({\sigma}_{d,i}^H\right)}^2,\end{array}} \end{equation*}

(5) \begin{equation*} {\displaystyle \begin{array}{c}{f}_{d,j}^{atac}=1+2\mathit{\log}\left({\sigma}_{d,j}^{atac}\right)-{\left({\mu}_{d,j}^{atac}\right)}^2-{\left({\sigma}_{d,j}^{atac}\right)}^2,\end{array}} \end{equation*}

where ${\sigma}_{d,i}^H\left({\sigma}_{d,j}^{atac}\right)$ and ${\mu}_{d,i}^H\left({\mu}_{d,j}^{atac}\right)$ are the elements in the $d$-th row and the $i$-th ($j$-th) column of the ${\mathbf{Z}}_{\boldsymbol{\mu}}^{\boldsymbol{H}}\left({\mathbf{Z}}_{\boldsymbol{\mu}}^{\boldsymbol{atac}}\right)$ and ${\mathbf{Z}}_{\boldsymbol{\sigma}}^{\boldsymbol{H}}\left({\mathbf{Z}}_{\boldsymbol{\sigma}}^{\boldsymbol{atac}}\right)$, respectively.

### Label transferring for ATAC non-anchor cells

After graph embedding, label knowledge is propagated from the RNA cells to the ATAC anchor cells in hybrid graph, while non-anchor cells in the ATAC graph still lack cell labels. We then designed an alignment strategy by treating the ATAC anchor cells viewed by two graphs having the same representation, which yields the following loss function:


(6) \begin{equation*} {\displaystyle \begin{array}{c}{\mathcal{L}}_{ali}=\frac{1}{\left|\mathbb{A}\right|}\sum_{a\in \mathbb{A}}{\left\Vert{\mathbf{z}}_{\boldsymbol{a}}^{\boldsymbol{H}}-{\mathbf{z}}_{\boldsymbol{a}}^{\boldsymbol{a}\boldsymbol{tac}}\right\Vert}_1,\end{array}} \end{equation*}

where ${\mathbf{z}}_{\boldsymbol{a}}^{\boldsymbol{H}}$ and ${\mathbf{z}}_{\boldsymbol{a}}^{\boldsymbol{a}\boldsymbol{tac}}$ are the feature vectors of the ATAC anchor cell $a$ in ${\mathbf{Z}}^{\boldsymbol{H}}$ and ${\mathbf{Z}}^{\boldsymbol{atac}}$, respectively; ${\left\Vert \ast \right\Vert}_1$ is the Euclidean norm. Therefore, the networks can be collaboratively trained by using not only the gene-level information in ${\mathbf{G}}^{\boldsymbol{H}}$ but also the peak-level information in ${\mathbf{G}}^{\boldsymbol{atac}}$.

### Graph reconstruction

To deal with the high sparsity in ${\mathbf{G}}^{\boldsymbol{H}}$ and ${\mathbf{G}}^{\boldsymbol{atac}}$, we combined them to one graph ${\mathbf{G}}^{\boldsymbol{M}}\in{\mathbb{R}}^{N\times N}$ and reconstructed a new graph ${\hat{\mathbf{G}}}^{\boldsymbol{M}}\in{\mathbb{R}}^{N\times N}=\sigma \left({\left({\mathbf{Z}}^{\boldsymbol{M}}\right)}^T{\mathbf{Z}}^{\boldsymbol{M}}\right)$, where $N=n_1+n_2$ is the total numer of cells in both graphs; $\sigma \left(\cdot \right)$ is the logistic sigmoid function and ${\mathbf{Z}}^{\boldsymbol{M}}\in{\mathbb{R}}^{k\times N}$ is the concatenation of ${\mathbf{Z}}^{\boldsymbol{H}}$ and ${\mathbf{Z}}^{\boldsymbol{atac}}$. ${\hat{\mathbf{G}}}^{\boldsymbol{M}}$ was supposed to be more informative than ${\mathbf{G}}^{\boldsymbol{M}}$, as it better illustrated the correlation between RNA cells and ATAC cells (Supplementary Note 4). The graph reconstruction loss is optimized using the binary cross entropy:


(7) \begin{equation*} {\displaystyle \begin{array}{c}{\mathcal{L}}_2=-\frac{1}{N}\sum_i^{N}\sum_j^{N}\left({\text{G}}_{i,j}^M\mathit{\log}\left({\hat{\text{G}}}_{i,j}^M\right)+\left(1-{\text{G}}_{i,j}^M\right)\mathit{\log}\left(1-{\hat{\text{G}}}_{i,j}^M\right)\right)\!,\\ \end{array}} \end{equation*}

where ${\text{G}}_{i,j}^M$ and ${\hat{\text{G}}}_{i,j}^M$ are the elements in the *i*-th row and the *j*-th column of ${\mathbf{G}}^{\boldsymbol{M}}$ and ${\hat{\mathbf{G}}}^{\boldsymbol{M}}$, respectively.

### Loss functions in HyGAnno

We finally applied softmax activation on ${\mathbf{Z}}^{\boldsymbol{M}}$ to compute the label indicator matrix ${\hat{\mathbf{Z}}}^{\boldsymbol{M}}= softmax\left({\mathbf{Z}}^{\boldsymbol{M}}\right)\in{\mathbb{R}}^{k\times N}$ for both reference and target cells. The cell label predictions of reference cells are optimized using cross entropy as follows: 


(8) \begin{equation*} {\displaystyle \begin{array}{c}{\mathcal{L}}_{rna}=-\frac{1}{n_1}{\sum}_{c\in \mathbb{Y}}{\sum}_{f=1}^k{\text{Y}}_{fc}\mathit{\log}\left(\ {\hat{\text{Z}}}_{fc}^M\right),\end{array}} \end{equation*}

where $\mathbb{Y}$ is the reference cell set, $\mathbf{Y}\in{\mathbb{R}}^{k\times{n}_1}$ contains the true labels of cells in $\mathbb{Y}$ and ${\text{Y}}_{fc}=1$ indicates that the cell *c* has the $f$-th label and ${\text{Y}}_{fc}=$0 indicates that the cell is not of this label. The final predicted label for target cell $i$ is determined as the $f$-th label when ${\hat{Z}}_{fi}^M\ge 0.5$. Along with other functions, the final loss function used in the HyGAnno framework is given as follows:


(9) \begin{equation*} {\displaystyle \begin{array}{c}\mathcal{L}={\mathcal{L}}_1+{\mathcal{L}}_2+\lambda{\mathcal{L}}_{ali}+{\mathcal{L}}_{rna},\end{array}} \end{equation*}

where $\lambda$ is a parameter adjusting the strength of the alignment loss, set to 1 as the default.

### Prediction reliability

Given an ATAC cell $c$, to describe its prediction reliability, we calculated the edge density and weight of cell $c$ in the reconstructed reference–target cell graph, as follows:


(10) \begin{equation*} {\displaystyle \begin{array}{c}{d}_c^k=\frac{e_c^k}{\left|\mathbb{K}\right|},{w}_c^k=\frac{\sum_{j\in \mathbb{K}}{\hat{\text{G}}}_{c,j}^M}{e_c^k}\end{array}} \end{equation*}

where $k$ is the label of the RNA cell group $\mathbb{K}$; ${e}_c^k$ is the edge number between cell $c$ and group $\mathbb{K}$; and ${\hat{\text{G}}}_{c,j}^M$ is the edge weight of the ATAC cell $c$ and RNA cell $j$. When ${e}_c^k=0$, ${w}_c^k$ is treated as 0. These formulas yield two vectors: ${\boldsymbol{d}}_{\boldsymbol{c}}=\left\{{d}_c^1,{d}_c^2,\dots, {d}_c^k\right\}$ and ${\boldsymbol{w}}_{\boldsymbol{c}}=\left\{{w}_c^1,{w}_c^2,\dots, {w}_c^k\right\}$. Together with vector ${\hat{\boldsymbol{z}}}_{\boldsymbol{c}}=\left\{{\hat{z}}_c^1,{\hat{z}}_c^2,\dots, {\hat{z}}_c^k\right\}$, which is the *c*-th column in the label indicator matrix ${\hat{\mathbf{Z}}}^{\boldsymbol{M}}$, if all ${\hat{\boldsymbol{z}}}_{\boldsymbol{c}},{\boldsymbol{d}}_{\boldsymbol{c}}$ and ${\boldsymbol{w}}_{\boldsymbol{c}}$ achieve their highest values at their $k$*-*th elements, the prediction of cell $c$ is confident; otherwise, it is ambiguous. That is, from the perspective of label probability, the label $k$ with the highest ${\hat{z}}_c^k$ is the prediction result for the cell $c$; meanwhile, from the perspective of graph connectivity, the highest ${\boldsymbol{d}}_{\boldsymbol{c}}^{\boldsymbol{k}}$ and ${\boldsymbol{w}}_{\boldsymbol{c}}^{\boldsymbol{k}}$ reflect that the cell $c$ is also densely connected to the RNA cell cluster with the label $k$. The consistency and inconsistency among these two perspectives are leveraged to identify confident and ambiguous predictions, respectively.

### Comparison with related work

In comparison to previous works in the field, our proposed method, HyGAnno, offers several unique advantages: (1) compared with Seurat v3 [15], scJoint [16], scGCN [17] and Conos [18], which take scRNA-seq as a reference and train model with only gene-level features, HyGAnno introduces peak-level information to the training process of the graph neural network since the epigenome pattern is also important for cellular heterogeneity. In addition, Seurat v3, Conos and scJoint failed to provide a stable function to detect unreliable predictions. Although scGCN provides two metrics to quantify the density of the predicted cell labels in the reference–target graph, the threshold setting of the proposed metrics is hard to decide. To solve this problem, HyGAnno applies the reconstructed graph and designed a parameter-free strategy to describe the prediction reliability and packaged corresponding function in our source codes. Furthermore, the usage of original peak features allows HyGAnno to screen cell type–specific peaks, which contributes to the research of investigating the cell type–specific gene regulatory mechanisms; (2) Compared with EpiAnno [23] and Cellcano [22], which take scATAC-seq as a reference, the complementary gene-level information used by HyGAnno may also improve the performance. Besides, both methods depend on high-quality labeled scATAC-seq reference, which is not commonly accessible. Incorporating advanced algorithms and numerous public scRNA-seq reference data, HyGAnno makes cell annotation for scATAC-seq more accurate and convenient.

### Dataset preparation

For the RNA-referenced methods, mouse brain data (GSE126074) were downloaded from the National Center for Biotechnology Information Gene Expression Omnibus (NCBI GEO) database and processed using Seurat and Signac, to create the input matrices ${\mathbf{X}}^{\boldsymbol{rna}}$, ${\mathbf{X}}^{\boldsymbol{atac}}$ and ${\mathbf{X}}^{\boldsymbol{gam}}$. The mouse lung data were prepared from *Tabula Muris* [26], and the atlas of the adult mice chromatin accessibility [27] was processed by Cicero to create ${\mathbf{X}}^{\boldsymbol{gam}}$. The input matrices of human PBMC and BMMC were downloaded from a previous study [1]. For the ATAC-referenced methods, GSE139369 and GSE129785 were downloaded from NCBI GEO and processed using Cicero to create ${\mathbf{X}}^{\boldsymbol{gam}}$ (Supplementary Note 5). The scRNA-seq (GSE176078) and scATAC-seq (GSE198639) datasets for breast cancer data were collected, and ${\mathbf{X}}^{\boldsymbol{gam}}$ was created using Signac (Supplementary Note 6). All data descriptions are shown in Supplementary Table 2.

### Cell embedding generation and visualization

For PCA, the first 30 principal components of the gene activity matrix were used for cell embeddings. For LSI, singular value decomposition (SVD) was applied to the term frequency-inverse document frequency (TF-IDF)-coded peak matrix, with the 2nd~30th SVD components serving as cell embeddings. For HyGAnno, scJoint and scGCN, the latent spaces in the neural networks were treated as cell embeddings (Supplementary Note 7).

### Evaluation metrics

The predictive performance was evaluated in terms of accuracy (ACC), normalized mutual information (NMI) [28] and F1 score. The F1 score was also weighted by the size of cell types in each dataset. The silhouette width (SW) score [29, 30] quantifies the capacity of a method to generate informative cell embeddings with ground truth cell types (Supplementary Note 8).

### Downstream analysis

Details of transcription factor (TF) motif enrichment, detection of cell type–specific peaks, trajectory analysis and copy number variation (CNV) calculation can be found in Supplementary Note 9.

## RESULTS

### The HyGAnno framework

**Figure 1:**
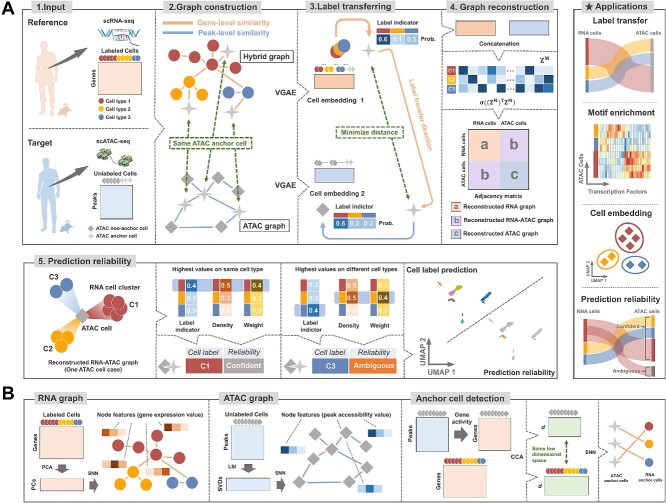
Overview of the HyGAnno framework. (**A**) The input of HyGAnno consists of an unlabeled peak matrix from scATAC-seq data and a well-labeled gene expression matrix from scRNA-seq data. HyGAnno is designed to train a model with both gene-level and peak-level features. For gene-level features, HyGAnno constructs a hybrid graph containing labeled cells in scRNA-seq (termed RNA cells) and cells highly correlated with RNA cells in scATAC-seq (termed ATAC anchor cells). For peak-level features, an ATAC graph is constructed containing all cells in scATAC-seq (termed ATAC cells). Then, HyGAnno embeds graphs with parallel variational graph auto-encoders and aligns the embedding of ATAC anchor cells viewed by two graphs. In this process, the cell labels are transferred from the RNA cells to ATAC cells. Meanwhile, a more informative RNA-ATAC cell graph can be constructed. When an ATAC cell in the reconstructed graph shows the highest connectivity to the cell type consistent with the label indicator, the prediction reliability is confident, otherwise, ambiguous. (**B**) SNN strategy followed by PCA (LSI) is applied on the low dimensional space of the gene expression matrix (peak matrix) to construct an RNA (ATAC) graph. The gene expression matrix and the gene-level transformation of the peak matrix are projected to a shared space using the CCA approach. A pair of cells from different modalities are detected and noted as anchor cells using SNN. Prob., probability; TF, transcription factor; VGAE, variational graph auto-encoder; SNN, shared nearest neighbor; PCA, principal component analysis; LSI, latent semantic indexing; CCA, canonical correlation analysis.

HyGAnno predicts cell labels for scATAC-seq data by aligning the embeddings of the same ATAC cells viewed in different graphs (Figure 1). First, HyGAnno builds a hybrid graph by computing the similarity of gene expression and gene activity features (collectively termed gene-level features) between RNA cells and ATAC cells. ATAC cells showing similar gene-level similarity with RNA cells (termed ATAC anchor cells) remain in the hybrid graph. Similarly, an ATAC graph can be constructed by computing the similarity of genome-wide chromatin accessible peak features (termed peak-level features) among ATAC cells. Then, HyGAnno employs parallel graph neural networks to embed hybrid and ATAC graphs into separate latent spaces and minimizes the distance between the embeddings of the same ATAC anchor cells in the two graphs. Using this approach, cell labels can be automatically transferred from scRNA-seq data to scATAC-seq data. In addition, HyGAnno reconstructs a consolidated reference–target cell graph that shows more complex graph structures, thus inspiring us to describe ambiguous predictions based on abnormal target–reference cell connections. Besides ensuring precise cell annotations, HyGAnno is also expected to outperform existing methods in visualizing interpretable cell clusters, handling noise cell labels in reference data and discovering cell type–specific peaks.

### Comparative annotation performance of HyGAnno with benchmarking methods

**Figure 2:**
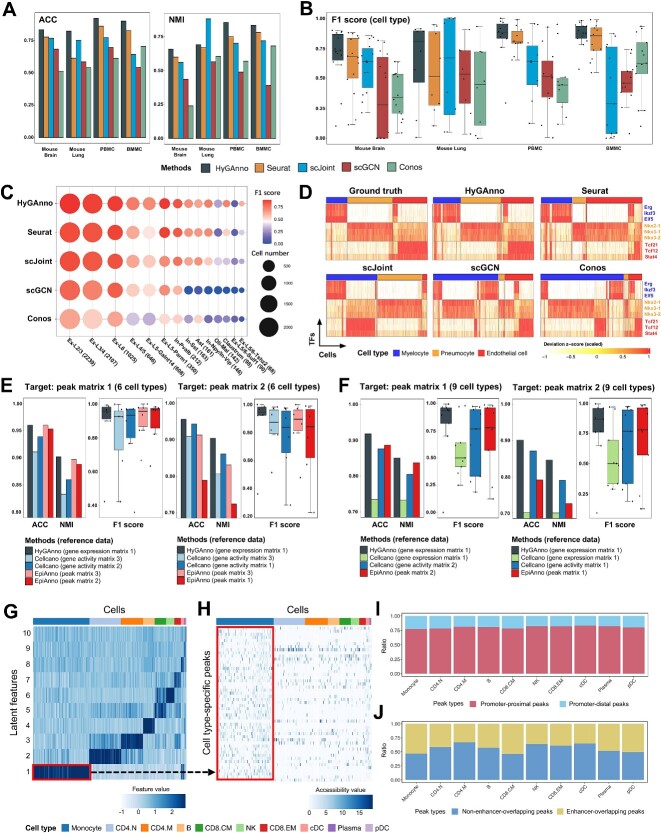
Comparative analysis of HyGAnno with RNA-referenced and ATAC-referenced benchmarks. (**A**) General cell annotation comparisons of HyGAnno, Seurat, scJoint, scGCN and Conos, evaluated in terms of ACC and NMI, on four target scATAC-seq datasets of mouse brain (8055 cells with 14 types), mouse lung (7499 cells with 8 types), PBMC (7828 cells with 10 cell types) and BMMC (14753 cells with 13 types). (**B**) Annotation performance for each cell type was evaluated in terms of F1 scores. Inside the boxes, dot points represent the F1 scores for different cell types. The top, middle and bottom lines of the box mark the 75th, 50th and 25th percentiles, respectively. Whiskers extend to data points within 1.5 times the interquartile range. (**C**) Comparisons of F1 scores regarding cell number of each cell type in mouse brain data. The brackets indicate the cell number of each cell type. (**D**) Activity scores of the transcription factor motifs were calculated using chromVAR, based on the prediction result of each benchmark. (**E**) Cell type prediction comparisons of HyGAnno, Cellcano and EpiAnno were evaluated in terms of the ACC, NMI and F1 scores. Two peak matrices containing 2588 cells and 3570 cells were used as the target. Six broad cell types were used for evaluation. (**F**) Same target data with nine cell types. (**G**, **H**) Heatmap of the latent space and the peak count matrix, where rows are cell type–specific features and cell type–specific peaks, respectively; columns are cells colored by ground truth. (**I**) Cell type–specific peak annotation based on their genome locations. (**J**) Overlapped cell type–specific enhancers with prompter-distal peaks. ACC, Accuracy; NMI, normalized mutual information; PBMC, peripheral bone mononuclear cell; BMMC, bone marrow mononuclear cell.

We first benchmarked HyGAnno against Seuratv3 [15], scJoint [16], scGCN [17] and Conos [18], which utilized scRNA-seq reference data. These methods were applied to mouse brain and lung datasets, as well as human peripheral blood mononuclear cell (PBMC) and bone marrow mononuclear cell (BMMC) datasets. Performance was measured in terms of ACC and NMI. Overall, HyGAnno achieved the highest average ACC (0.87) among the four datasets (Figure 2A), followed by Seurat (0.77) and scJoint (0.73). Even though scGCN also takes advantage of the GCN algorithm, the ACC and NMI are significantly lower than HyGAnno because it trains models without reference graphs.

To better assess the performance of the imbalanced datasets, we calculated the F1 scores for each cell type according to different benchmarks (Figure 2B). HyGAnno achieved a better performance for most cell types across four datasets. In particular, we confirmed that HyGAnno had a higher predictive resolution for rare cell types (Figure 2C). For example, Claustrum and Ex-L5/6-Tshz2 in mouse brain data contained only tens of cells. In addition, even when the cell number of the reference (2623) was twice as small as that of the target (7499) in the mouse lung data, unlike other methods that struggle with insufficient training, HyGAnno precisely annotated the cell types, e.g. endothelial cells (Supplementary Figure 1). Then, to investigate how cell labeling influences downstream analyses, we analyzed the enrichment of TFs in the accessibility peaks of the predicted ATAC cell types in mouse lung, using the deviation of *z*-scores calculated by chromVAR [31]. We assessed the enrichment patterns of the cell type–specific TFs, such as *Erg*, *Nkx2-1* and *Tcf21* [32–35] based on the ground truth and predicted cell labels of myelocytes, pneumocytes and endothelial cells. The TF motif enrichment patterns based on the predictions of HyGAnno were highly similar to those of the ground truth (Figure 2D), which highlights how accurate annotation of HyGAnno can ensure downstream analysis.

Next, for comprehensive comparisons, we benchmarked HyGAnno with Cellcano [22] and EpiAnno [23], which utilize the cell labels of the scATAC-seq reference data. To assess the prediction results of HyGAnno, Cellcano and EpiAnno, we used fixed target data, but different reference data. First, we checked the annotation results for six cell types. HyGAnno performed similar performance to EpiAnno and Cellcano (Figure 2E). Then, to assess the annotation ability of more cell types, we increased the number of cell types to nine, to include more subtypes based on the original research [1]. We observed the pronounced superiority of HyGAnno in achieving high-resolution cell annotation, with a median of F1 scores of 0.86, much higher than those for both Cellcano and EpiAnno (0.75) (Figure 2F). Finally, to examine whether the performance of Cellcano could be improved, we trained Cellcano using the same scRNA-seq input as HyGAnno. However, the results worsened (indicated by the second boxes in Figure 2F), suggesting that HyGAnno was more capable of exploiting high-quality reference data. We also demonstrated the computational efficiency of HyGAnno with all compared methods in Supplementary Figure 2, where HyGAnno shows affordable running time and memory requirement.

As HyGAnno directly trains the model with genome-wide accessible peak (peak-level) features (Supplementary Figure 3), we can extract the optimized weight matrices from deep neural networks to interpret the importance of each peak-level feature on cell type identification. We trained a one-layer network for PBMC data, where cell type–specific latent features (Figure 2G) were directly connected to the input peak-level features. This configuration enables the efficient identification of cell type–specific peaks (Figure 2H). Given that the cellular specificity is largely governed by CREs across entire genome sequences, we annotated cell type–specific peaks according to their proximity to transcription start sites (Figure 2I). We found that around 25% of these peaks were promoter-distal, indicating the importance of distal intergenic regions in distinguishing cell types. To further investigate the CREs in these promoter-distal peaks, we mapped them to scEnhancer [36], a single-cell enhancer database, and found that half of these peaks were overlapped with cell type–specific enhancers (Figure 2J). We also conducted the same experiment on BMMC data. The detected cell type–specific peaks got a higher overlapping ratio on cell type–specific enhancers than the ratio of randomly selected peaks, indicating high interpretability of our proposed model (Supplementary Figure 4). All these results proved the powerful ability of HyGAnno in extracting cell type–specific characteristics by giving weights to not only the promoter-proximal regions but also the CREs in distal intergenic regions, thus guaranteeing the precision of the high-resolution cell-type annotation.

### Leveraging peak-level features improves cell embeddings

**Figure 3:**
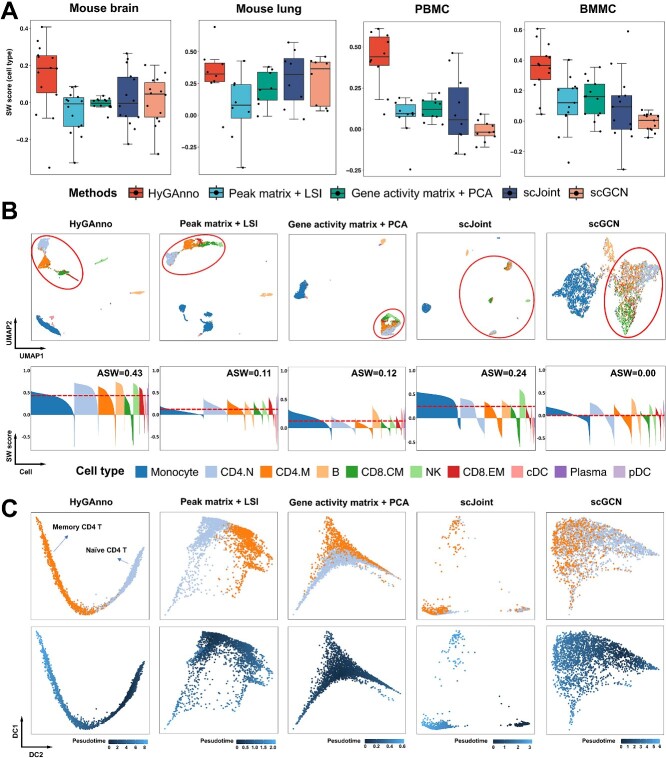
Evaluation of cell embeddings. (**A**) SW scores of the cell embeddings obtained from HyGAnno, LSI, PCA, scJoint and scGCN. Inside the boxes, dot points represent SW scores for different cell types. The top, middle and bottom lines of the box mark the 75th, 50th and 25th percentiles, respectively. Whiskers extend to the data points that are within 1.5 times the interquartile range. (**B**) UMAP plots (upper row) and the corresponding SW score (bottom row) of PBMC cell embeddings. The cells are colored based on ground truth cell types. The four T cell subtypes are surrounded by solid circles. (**C**) Trajectory analysis based on the cell embeddings of CD4.N cells and CD4.M cells. The cells are colored based on ground truth cell types (upper row) and pseudotime calculated using DPT (bottom row). UMAP, uniform manifold approximation and projection; SW, silhouette width; DPT, diffusion pseudotime.

To investigate the effect of peak-level information on cell embeddings, we generated cell embeddings using five methods: HyGAnno, LSI [20], PCA, scJoint and scGCN. We assessed these methods by calculating the SW scores for the embedding spaces across various datasets (Figure 3A). Notably, HyGAnno outperforms PCA, scJoint and scGCN since it leverages peak-level features in the training process. The small interquartile range of HyGAnno indicates its high robustness. LSI also considers peak-level features; however, its performance is limited owing to the lack of scRNA-seq reference data.

To interpret the cell embeddings, we then visualized the cell embeddings of the PBMC data across different methods using uniform manifold approximation and projection (UMAP) [37] (Figure 3B). Taking the T-cell subtypes as an example, we observed that HyGAnno, LSI, PCA and scJoint could distinguish the four T cell subtypes (solid circles in Figure 3B) from B cells and monocytes. In contrast, scGCN embedded all T cell subtypes together, creating a mixture that resulted in the lowest average silhouette width (ASW) score. Both HyGAnno and LSI captured the differentiation processes from naïve CD4 T cells (CD4.N) to memory CD4 T cells (CD4.M) [38]. However, HyGAnno achieved a higher ASW score (0.43) than LSI (Figure 3B) and separated natural killer (NK) cell cluster by taking the cell embedding information from reference data (Supplementary Figure 5A). Although scJoint detected the discrete cell islands, it exhibited a lower ASW score (0.24) than that of HyGAnno. This result indicated a misunderstanding of cell development and differentiation information led by scJoint forcing the cells with the same predicted labels to be near the random centers.

To further demonstrate the strength of the cell embedding obtained from HyGAnno in cellular differentiation analysis, we calculated the pseudotime [39] of CD4.N and CD4.M based on different cell embedding strategies (Figure 3C) since CD4.N can differentiate into effector cells upon exposure to antigen to clear the infection and the surviving cells become CD4.M [38]. We found that the cell embedding of HyGAnno is the only one achieving continuous trajectory to show this important differentiation process. In addition to the PBMC data, the UMAP visualization of BMMC also suggests the interpretable cell embeddings (Supplementary Figure 5B and C); for instance, the differentiation trend from common lymphoid progenitors to precursors of lymphocytes (Pre. B cells) and B cells [40] (Supplementary Figure 5D). Collectively, these results highlighted the collaborative training of HyGAnno using both gene-level features from the scRNA-seq reference and peak-level features from the scATAC-seq target data, which provides interpretable cell embeddings, ensuring the trajectory analysis and describing cellular similarity and potential differentiation process.

### HyGAnno resists noisy cell labels in reference data

**Figure 4:**
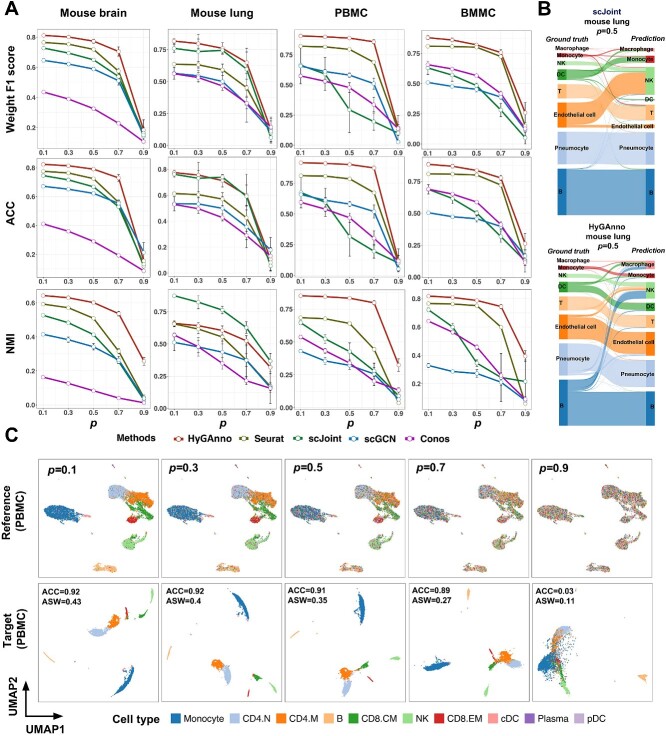
Evaluation of HyGAnno against noisy reference data. (**A**) Weighted F1 scores, ACC and NMI were calculated for HyGAnno, Seurat, scJoint, scGCN and Conos across four datasets with increasing noise level *p* from 0.1 to 0.9. Each point indicates the mean value among 10 randomly generated cell label lists and the whisker is extended by standard deviation. (**B**) Sankey plots indicating the details of how cell labels are transferred from reference to target data of HyGAnno and scJoint under the condition of *P* = 0.5 in mouse lung data. (**C**) UMAP plots of PBMC reference data colored by noise labels (upper row) and PBMC target data colored by predicted labels (bottom row), with *p* from 0.1 to 0.9.

Most scRNA-seq data are manually annotated and adjusted by exploring the expression patterns of the marker genes, which inevitably leads to incorrect cell labels. To assess how noisy cell labels in scRNA-seq references affect the cell type annotation, we randomly generated 10 false cell lists for each data, by increasing the noise level proportion (*p*) from 0.1 to 0.9. In each proportion, the average performance of the five methods across the four datasets was reported in terms of weighted F1 score, ACC and NMI (Figure 4A).

We found that all methods deteriorated as *p* increased and finally lost their annotation ability when *p* reached 0.9 (Figure 4A). HyGAnno outperformed other methods by consistently achieving the highest weighted F1 and ACC scores for all data. Meanwhile, Seurat was the second-best performer, with a similar performance to HyGAnno in the mouse brain and BMMC data. In the mouse lung data, scJoint had higher NMI scores than HyGAnno. This discrepancy arises because a high NMI score is obtained if two samples of the same class are classified into the same group. Hence, a higher NMI paired with a relatively lower weighted F1 score indicates that scJoint might incorrectly annotate the entirety of some cell clusters. To verify this, we illustrated the prediction results of HyGAnno and scJoint using Sankey plots under the condition of *p* = 0.5 in mouse lung data (Figure 4B). We observed that scJoint failed to annotate whole clusters of monocytes, dendritic cells and endothelial cells, in which most endothelial cells were labeled as NK cells, despite the huge gap in biological functions between them. In contrast, although some B cells were wrongly labeled as NK cells by HyGAnno, the prediction was still acceptable, as there were no entirely mislabeled cases in any clusters. We also visualized the reference and target data of PBMCs with *p* from 0.1 to 0.9 using UMAP (Figure 4C). We found that before *p* reached 0.9, not only was the ACC of HyGAnno almost unchanged from 0.92 to 0.89 but the cell embedding structures was also preserved, with an ASW score of 0.27. The ability of HyGAnno to withstand the impact of noisy cell labels in reference data is largely attributed to its graph generative mechanism. Specifically, when dissimilar cells are mistakenly clustered together due to noisy labels, the resulting reconstructed graph significantly diverges from the initial one, leading to a notable increase in graph reconstruction loss, which can be effectively optimized by the model.

### HyGAnno describes the reliability of predicted cell labels

**Figure 5:**
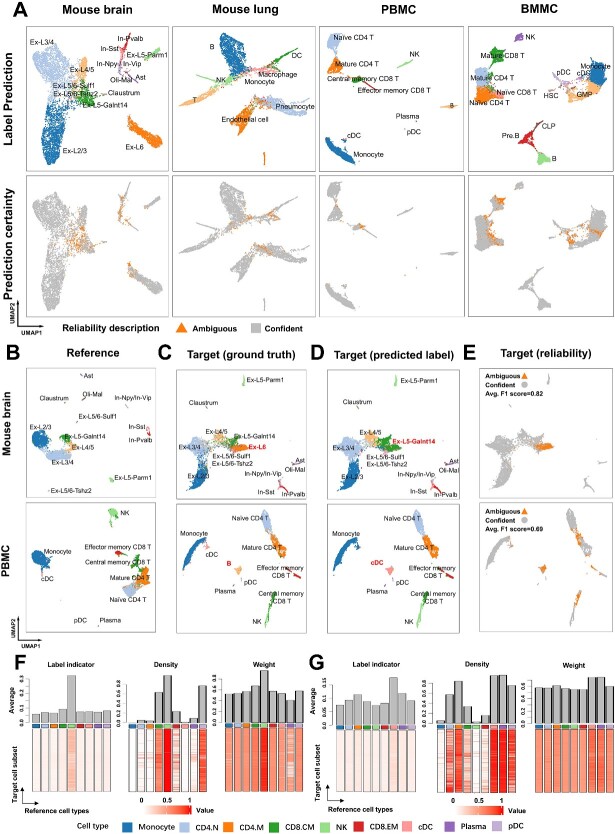
Description of prediction reliability based on the reconstructed graph. (**A**) Cell type annotation and the prediction reliability of four data. (**B**) UMAP plot of the reference data of mouse brain and PBMC, excluding the Ex-L6 cells and B cells, respectively. Cells are colored based on their ground truth labels. (**C**–**E**) UMAP plot of the target data of mouse brain and PBMC, including Ex-L6 cells and B cells. Cells are colored based on their ground truth labels, prediction labels and prediction reliability, respectively. (**F**, **G**) Heatmaps of label indicator, density and weight matrix obtained from the reconstructed graph of PBMC data.

To assess how HyGAnno handles ambiguous predictions, we first reconstructed reference–target cell graphs using HyGAnno from four datasets: mouse brain, mouse lung, PBMC and BMMC. We quantified the differences between the reconstructed and initial graphs based on the density of linked edges (DLEs) between RNA and ATAC cells. Based on the heatmaps shown in Supplementary Figure 6, the reconstructed graph showed more non-diagonal DLE values than the initial graph, indicating that the reconstructed graph could amplify the abnormal cell–cell connections hidden in the initial one. This characteristic inspired us to design the strategy (Material and methods) to detect ambiguous predictions. We found that the ambiguous predictions of the above four datasets are typically scattered within the regions with highly similar cell types (Figure 5A); for instance, a clear cell boundary between the clusters of granulocyte–monocyte progenitors (GMPs) and monocytes in the BMMC data. Because these ambiguously predicted cells may be unreliable, we eliminated them from the datasets and observed notable improvements across several evaluation metrics (Supplementary Figure 7). This observation suggested the removal of the cells in target data with ambiguous predictions.

However, the ambiguous predictions are caused not only by the high cell-type similarity in the target data but also by the cell-type bias between the reference data and target data. In addition to the detection of ambiguous predictions for the non-biased datasets mentioned above, we next simulated the scenarios of cell type–biased datasets. We manually removed Ex-L6-Tle4 cells and B cells from the reference mouse brain data and PBMC data, respectively (Figure 5B), while the target data remained unchanged (Figure 5C). After predicting cell labels, we compared results with the ground truth and found that most Ex-L6 cells and B cells were inappropriately labeled as Ex-L5-Galnt14 cells and conventional dendritic cell (cDC) cells, respectively (Figure 5D). However, the clusters of these two cell types were successfully captured by the ambiguous predictions, with high F1 scores of 0.82 and 0.69 (Figure 5E). We also noticed that the ambiguous predictions dominated the entire of their cell clusters, which could be treated as a visual characteristic to distinguish the scRNA-seq reference lacking cell types. Hence, we suggested users made their own choice to remove cells with ambiguous predictions from downstream analysis or change to another scRNA-seq reference with enough cell types.

Finally, to further demonstrate how the prediction reliability is denoted, we used the above cell type–biased PBMC data as an example and visualized the label indicator, density and weight matrices (Figure 5F and G), which helped us formulate the pattern of the abnormal cell–cell connections amplified by the reconstructed graph. According to the label indicator matrix (Figure 5F), the target cell subsets (rows) had the highest values for the NK cell type (column); hence, they were predicted to be NK cells. Based on the density and weight matrices, which summarize the connection patterns in the reconstructed graph (Supplementary Figure 8A), we found that the target NK-like cells were densely connected to the reference NK cells. This consistent observation from two scopes suggests confident labeling prediction. On the contrary, for another target cell subset (Figure 5G), although most of them were predicted to be cDC, the inconsistent dense connection with not only the reference cDC but also plasma, plasmacytoid dendritic cell (pDC) and CD4.M cells lowered their prediction reliability (Supplementary Figure 8B). Collectively, as an additional procedure after HyGAnno cell annotation, the cell type prediction reliability can be described using the reconstructed graph. For user convenience, we have packaged the function to annotate the prediction reliability as one potential way to evaluate the reference data.

### HyGAnno annotates neoplastic and normal cells in breast cancer

**Figure 6:**
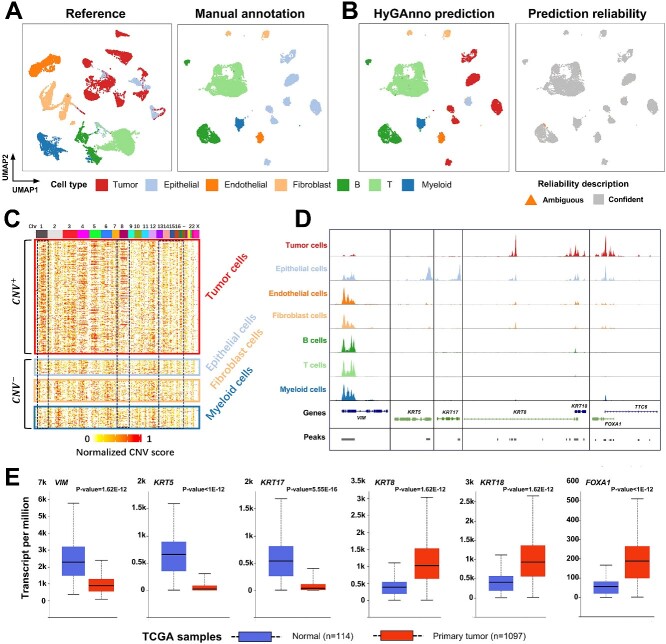
Evaluation of cell type prediction in the tumor microenvironment using HyGAnno. (**A**) UMAP plots of reference data and target data from breast cancer, colored based on manually annotated cell types. (**B**) UMAP plots of target data, colored based on HyGAnno cell type prediction and prediction reliability. (**C**) Normalized CNV patterns of each cell cluster. (**D**) Enrichment of differential accessibility peaks of tumor and normal cells in the promoter regions of potential signature genes. (**E**) Gene expression differences of observed genes between 1097 breast cancer and 114 normal samples.

Because of the shared lineages of tumor cells with their normal counterparts [41], it is challenging to identify them using marker genes or clustering analysis. To assess the capacity of HyGAnno to annotate cells within the tumor microenvironment (TME), we gathered scRNA-seq and scATAC-seq (Figure 6A) as reference and target datasets from breast cancer and annotated them using the same approach outlined in the original works [42, 43], in which manual cell annotation failed to differentiate tumor and epithelial cells in the scATAC-seq data. To address this, we leveraged cell labels of scRNA-seq data using HyGAnno and obtained new cell annotations, wherein immune, fibroblast and endothelial cells remained consistent with the manual annotation results, while partial epithelial cells were reannotated as tumor cells with high prediction reliability (Figure 6B). Because the ground truth of the scATAC-seq data was unclear, we presented the following evidence to support the reliability of the identified normal cells and tumor cells.

First, we demonstrated the differences in DNA amplification between predicted tumor cells and normal cells. Based on previous works [42, 44], we inferred the DNA CNV signals by calculating the normalized CNV scores of the scATAC-seq data (Figure 6C). We found that tumor cells have stronger CNV signals than the epithelial cells on chromosome 1, which has been reported to be involved in quantitative anomalies in 50~60% of breast cancers [45]. Because malignant transformation occurs predominantly in breast tissues from epithelial cells [46], we used the CNV patterns of fibroblasts and the infiltrating myeloid cells as the normal background. The predicted epithelial cells showed an amplification pattern similar to that of the normal background on each chromosome, indicating a high possibility of these cells being normal.

To further demonstrate the reliabililty of the HyGAnno prediction, we extracted the differential accessibility peaks between tumor cells and epithelial cells and checked whether they were enriched in the promoter regions of certain genes, as this might reflect the specific expression patterns of these two cell categories. The peaks of epithelial cells and tumor cells were notably enriched in the gene sets of ‘*VIM*, *KRT5*, *KRT17*’ and ‘*KRT8*, *KRT18*, *FOXA1*’, respectively (Figure 6D, Supplementary Figure 9A). However, only *VIM* and *KRT5* exhibited high gene expression levels in epithelial cells, owing to the limited sample amount (Supplementary Figure 9B). To clarify the differential expression patterns, we entered these genes into a well-established database, UALCAN [47], and compared their expression levels in 114 normal samples and 1097 breast cancer samples. The expression levels of *VIM*, *KRT5* and *KRT17* in normal samples were significantly higher than those in breast cancer samples (Figure 6E). Conversely, the expression levels of *KRT8*, *KRT18* and *FOXA1* showed opposite trends. This is consistent with results obtained from peak enrichment, thus providing compelling evidence that the predicted tumor cells and normal cells in the TME are precise and reasonable and demonstrating the suitability of HyGAnno for researchers who wish to annotate scATAC-seq derived from tumor tissues and explore the mechanisms of the carcinogenesis from an epigenomic perspective.

### The framework of HyGAnno shows high robustness

As HyGAnno has shown its superiority in scenarios mentioned above, we further conducted additional experiments to demonstrate the robustness of HyGAnno. First, we conducted ablation studies by modifying the original model into three cases: one with a single layer, one without the alignment loss function [Equation (6)] and one without the graph reconstruction loss function [Equation (7)]. Their performance according to mouse brain, mouse lung, PBMC and BMMC datasets was measured by ACC and NMI in Supplementary Figure 10. We found that both loss functions are essential for an accurate prediction while the number of layers affected the predictions on each dataset in different degrees. Then, we evaluated the overfitting issue of HyGAnno model through recording prediction ACC of both reference data and target data according to the increasing epoch (Supplementary Figure 11). We found that after about 100 epochs, both ACC curves tend to be converged and with no significant decrease in next 400 epochs, which indicates the ability of the model to avoid the overfitting issue. Lastly, we evaluated the robustness of HyGAnno according to different parameter settings. In the graph construction part, we found that HyGAnno was robust to parameters of the low-dimension number $n$ and shared $k$-nearest neighbors ${k}_1$ and ${k}_2$, which were set as 30, 5~25 and 5~25, respectively (Supplementary Figure 12A). Note that a high value of ${k}_2$ (20~25) is recommended when target data have a larger cell number than the reference data (Supplementary Table 3). In the model training part, although a high dimension number of embedding space $d$ improves the performance (Supplementary Figure 12B), we take the trade-off by setting $d$ = 128 and learning rate $l$ = ${10}^{-4}$ to accelerate the training process.

## DISCUSSION

scATAC-seq, one of the most important sequencing protocols, is used to study genome-wide open chromatin regions at the epigenetic level. Together with scRNA-seq at the transcriptomic level, scATAC-seq offers the opportunity to unveil the intricate regulatory mechanisms across diverse cellular phenotypes [1, 48]. As a prerequisite for single-cell multi-omics analysis, cell type annotation has been well explored in scRNA-seq data, while the same problem in scATAC-seq data has received less attention. This gap limits our understanding of the roles of CREs in determining cell type–specific functionalities, such as promoter-distal open chromatin regions, which contain enhancers that regulate cell type–specific transcription. To address this, we developed a deep graph–based model, HyGAnno, that transfers cell label information from scRNA-seq data to cells assayed by means of scATAC sequencing. In contrast to other cell type annotation methods that train models with only gene-level features, HyGAnno innovatively constructs advanced graph neural networks with both peak-level and gene-level features, thus exploring the possibility of modeling transcription processes with the assistance of multi-omics data.

According to the experimental results, HyGAnno outperformed most existing cell annotation tools, especially those that overlooked the promoter-distal accessible peak information during the model training process. The interpretable cell embedding of HyGAnno provided new insights into traditional trajectory analysis, and the continuous pseudotime may reveal the key genes dynamically expressed in a specific cell differentiation process. As a label transferring method, HyGAnno relies on the cell label information in the scRNA-seq reference data, which is manually or computationally annotated based on expression similarities to some marker genes. To reduce the effect of cell labels in reference, HyGAnno reconstructs a new graph to retain the backbone of the inter-modality and intra-modality cell connections in the initial graphs. Reconstruction loss can reduce the weight of the classification loss affected by noisy labels. Meanwhile, the reconstructed graph can also amplify the abnormal cell–cell connections in the initial graphs and hence, be leveraged to judge the prediction reliability by assigning each cell a confident or an ambiguous description. When ambiguous predictions appear in a boundary or a mixture consisting of multiple cell types, we suggest removing these predictions before downstream analysis. However, the selected scRNA-seq reference is suggested to be discarded if ambiguous predictions dominate entire cell clusters as this characteristic indicates a lack of cell labels in reference. We also validated the facility of HyGAnno in TME. HyGAnno successfully distinguished tumor cells and normal cells, thereby providing insights into the mechanisms of carcinogenesis from an epigenomic perspective. Finally, with the growing repository of high-quality scRNA-seq datasets, using scRNA-seq references that correspond to the tissue with the scATAC-seq target becomes more practical [49]. This development provided a critical foundation for HyGAnno to effectively transfer cell labels from scRNA-seq data to scATAC-seq data.

### Limitations and future work

Although HyGAnno shows higher ACC on cell label prediction tasks, its training process involves several intermediate steps that increase time and memory usage. We aimed to minimize this problem by extracting only key regions from entire graphs that may reduce the number of nodes and edges for training. Besides, like other label transferring methods, HyGAnno relies on high-quality reference data, which should be well annotated, large size and high resolution. We also plan to further extend HyGAnno to evaluate the quality of the reference dataset, thereby advising users on changing additional scRNA-seq references or maintaining the current one. In addition, HyGAnno has the potential to be adapted to multimodal data. Specifically, HyGAnno can train a model using multimodal data [50, 51] as the reference. By constructing multiple hybrid graphs and using ATAC-anchor cells as a bridge, HyGAnno can reinforce information-sharing among modalities, relieving low-throughput and sparse data from these joint profiling technologies [52, 53].

**Key Points**

- We proposed a computational approach that can efficiently identify cell types for various public single-cell ATAC-seq data, benefiting the biologists who desire to analyze single-cell omics data from an epigenomic level.
- HyGAnno directly takes genome-wide accessible peaks as input features to train the model, which can better reflect the cellular heterogeneity compared with other methods that only use gene features.
- HyGAnno has been tested on complex tissues, such as tumor microenvironment, showing facility on distinguishing neoplastic and normal cells in breast cancer.

## Supplementary Material

Supplementary_bbae152

## Data Availability

All data used in this study were obtained from public resources and can be downloaded from original publications. For user convenience, we provided all pre-processed datasets in a data repository called Figshare (https://doi.org/10.6084/m9.figshare.25133492.v1)*.* The codes and tutorials are freely available at https://github.com/WeihangZJP/HyGAnno, under an MIT license. Supplementary data are available online.

## Author Biographies

**Weihang Zhang** received his Matster degree in Computer Science from University of Tsukuba in 2022. He is currently a PhD student in the Department of Computational Biology and Medical Sciences, University of Tokyo. His research interests include machine learning and single-cell genomics.

**Yang Cui** received his Master degree in Medical Science from Tokyo Medical and Dental University in 2022. He is currently a PhD student in the Department of Computational Biology and Medical Sciences, University of Tokyo. His research interests include oncology, immunology, and bioinformatics.

**Bowen Liu** received his Master degree in Control Science and Engineering from Xi’an Jiaotong University in 2020. He is currently a PhD student in the Department of Computational Biology and Medical Sciences, University of Tokyo. His research interests include deep learning, data mining and bioinformatics.

**Martin Loza** is an assistant professor at the Institute of Medical Science, the University of Tokyo. His research focuses on computational analysis of epigenetics and genome biology.

**Sung-Joon Park** is an associate professor at the Institute of Medical Science, the University of Tokyo. His research focuses on computational genome biology.

**Kenta Nakai** received his PhD degree from Kyoto University in 1992. He is currently a full professor at the Human Genome Center, the Institute of Medical Science, the University of Tokyo, Japan. His main research interest is to develop computational methods for interpreting biological information, especially that of transcriptional regulation, from genome sequence data.
