# Supplementary material for: HyGAnno: hybrid graph neural network–based cell type annotation for single-cell ATAC sequencing data
Source: Brief Bioinform. 2024 Apr 5;25(3):bbae152. doi: 10.1093/bib/bbae152 (PMC10998639; doi:10.1093/bib/bbae152)
Supplement: Supplementary_bbae152 [file supplementary_bbae152.pdf]

Supplementary Information for:
HyGAnno: Hybrid graph neural network-based cell type annotation for single-cell ATAC sequencing data

Contents

Supplementary Notes **2**

Supplementary Note 1: Details in graph construction 2
Supplementary Note 2: Number of the anchor cells 4
Supplementary Note 3: Graph embedding by VGAE auto-encoders 5
Supplementary Note 4: Differences between the initial and reconstructed RNA-ATAC graph.. 7
Supplementary Note 5: Details of data preparation for benchmarking 9
Supplementary Note 6: Details of data preparation for tumor cell detection 11
Supplementary Note 7: Cell embedding generation methods 12
Supplementary Note 8: Evaluation metrics 13
Supplementary Note 9: Downstream analysis 15

Supplementary Figures **16**

Supplementary Figure 1 16
Supplementary Figure 2 17
Supplementary Figure 3 18
Supplementary Figure 4 19
Supplementary Figure 5 20
Supplementary Figure 6 22
Supplementary Figure 7 23
Supplementary Figure 8 24
Supplementary Figure 9 25
Supplementary Figure 10 26
Supplementary Figure 11 27
Supplementary Figure 12 28

Supplementary Tables **29**

Supplementary Table 1: Properties of the constructed graphs 29
Supplementary Table 2: Descriptions of datasets 30
Supplementary Table 3: Prediction performance according to different numbers of anchor cells 31

References **32**

Supplementary Notes

Supplementary Note 1: Details in graph construction

For the hybrid graph, it contains two kinds of edges: edges between RNA cells themselves and edges between RNA anchor cells and ATAC anchor cells. For the first kind of edges, we applied Principal Component Analysis (PCA) to project the gene feature space of $\mathbf{X}^{rna}$ to a low-dimensional space, which consists of top 30 PCs. Then, if any of the two RNA cells in this latent space are the shared k-nearest neighbors (SNNs), an edge is assigned to them. For the second kind of edges, we measured the similarity between cells in different modalities by transforming the peak matrix $\mathbf{X}^{atac}$ to the gene activity matrix (GAM) $\mathbf{X}^{gam} \in \mathbb{R}^{g \times n_2}$, which represents the degree of accessible gene body regions scored by the total number of mapped scATAC-seq reads[1]. Depending on different datasets, the GAMs may be directly provided from original works or be generated through other functions such as Signac and Cicero. After taking intersected genes of $\mathbf{X}^{rna}$ and $\mathbf{X}^{gam}$, we performed a similar standardization transform mentioned in scGCN on these two matrices and conducted Canonical Correlation Analysis (CCA) to project them into a low dimension space (30 dimensions in all experiments), where the projected data achieves the highest correlation coefficient. Any RNA and ATAC cells clustered together by SNN are assigned as RNA anchor cells and ATAC anchor cells for the corresponding clusters. Finally, we got the hybrid graph $\mathbf{G}^{H} \in \mathbb{R}^{(n_1+|\mathbb{A}|)\times(n_1+|\mathbb{A}|)}$ and its feature matrix $\mathbf{X}^{H} = [\widetilde{\mathbf{X}}^{rna},\ \widetilde{\mathbf{X}}^{gam}_{\mathbb{A}}] \in \mathbb{R}^{g\times(\mathrm{n}_1+|\mathbb{A}|)}$, where $G^{H}_{ij} = G^{H}_{ji} = 1$ stands the edge of the cell i and j; $|*|$ represents the size of a set; $\mathbb{A}$ is the set of ATAC anchor cell; $\widetilde{\mathbf{X}}^{rna} \in \mathbb{R}^{g\times n_1}$ and $\widetilde{\mathbf{X}}^{gam}_{\mathbb{A}} \in \mathbb{R}^{g\times|\mathbb{A}|}$ are the whole and subset of the standardized feature matrix of $\mathbf{X}^{rna}$ and $\mathbf{X}^{gam}$, respectively.

For the ATAC graph, a term frequency-inverse document frequency (TF-IDF) transformation followed by Singular Value Decomposition (SVD) is performed to X^{atac} to project it into a space with 30 dimensions. This strategy is also known as the Latent Semantic Indexing (LSI). We removed the first dimension as it might capture the technical variation rather than the biological variation. Similarly, we conducted SNN strategy on the LSI latent space with remained 29 components, obtaining the ATAC graph $\mathbf{G}^{atac} \in \mathbb{R}^{n_2\times n_2}$. To train HyGAnno with

peak-level information, instead of the frequently-used gene-level matrix of $\mathbf{X}^{gam}$, the peak-level matrix of $\widetilde{\mathbf{X}}^{atac} \in \mathbb{R}^{p \times n_2}$ is treated as the node feature matrix of the ATAC graph, where $\widetilde{\mathbf{X}}^{atac}$ is the TF-IDF normalized matrix of $\mathbf{X}^{atac}$.

Supplementary Note 2: Number of the anchor cells

Working as bridges between the RNA graph and ATAC graph, anchor cells affect the cell annotation performance a lot. In our research, instead of selecting an exact number of anchor cells, we applied shared k_2-nearest neighbor strategy within the shared space of RNA and ATAC modalities, that is, for a cell a in reference data and another cell b in target data, they are assigned as anchor cells if and only if cell a is in the k_2-nearest neighbor of cell b, meanwhile cell b is in the k_2-nearest neighbor of cell a. Hence, the selection of the number k_2 is of vital importance to the number of anchor cells. To figure out how k_2 can decide the prediction performance, we conducted experiments based on various setting of k_2 and recorded the numbers of detected anchor cells together with the numbers of edges among anchor cells in Supplementary Table 3. When k_2 was set from 5 to 25, we found that there is no significant performance gap in mouse brain and PBMC datasets. However, the performance of BMMC and mouse lung datasets peaked at a relatively high value of k_2 (20~25). We assumed that this might be because the cell number of target data in BMMC and mouse lung datasets are larger than that of reference data, which easily causes insufficient connections between target and reference data. To enhance the connections, a higher k_2 is thus needed, and the final number of anchor cells depends on practically used data.

Supplementary Note 3: Graph embedding by VGAE auto-encoders

Regarding each of the hybrid graph and the ATAC graph, we embed it into a k-dimensional space. In each space, to satisfy the loss function striction of the variational graph auto-encoder (VGAE)[2] , the cell embedding should be hypothetically sampled from a specific k-multivariate normal distribution with independent variables:

$$q(\mathbf{Z}^{\boldsymbol{H}}|\mathbf{X}^{\boldsymbol{H}},\mathbf{G}^{\boldsymbol{H}}) = \prod_{i=1}^{k} \mathcal{N}\left(\boldsymbol{z}_i^{\boldsymbol{H}} \middle| \boldsymbol{\mu}_i^{\boldsymbol{H}}, diag(\boldsymbol{\sigma}_i^{\boldsymbol{H}})\right),$$

$$q(\mathbf{Z}^{\boldsymbol{atac}}|\widetilde{\mathbf{X}}^{\boldsymbol{atac}},\mathbf{G}^{\boldsymbol{atac}}) = \prod_{j=1}^{k} \mathcal{N}\left(\boldsymbol{z}_j^{\boldsymbol{atac}} \middle| \boldsymbol{\mu}_j^{\boldsymbol{atac}}, diag(\boldsymbol{\sigma}_j^{\boldsymbol{atac}})\right)$$

where $\mathbf{Z}^{\boldsymbol{H}} \in \mathbb{R}^{k\times(n_1+|\mathbb{A}|)}$ and $\mathbf{Z}^{\boldsymbol{atac}} \in \mathbb{R}^{k\times n_2}$ are the embeddings of graph $\mathbf{G}^{\boldsymbol{H}}\ and\ \mathbf{G}^{\boldsymbol{atac}}$, respectively; k is the number of the cell types in reference data; $\boldsymbol{\mu}_i^{\boldsymbol{H}}$ and $\boldsymbol{\sigma}_i^{\boldsymbol{H}}$, which are k-dimensional vectors, represent the mean and covariance of the multivariate normal distribution, respectively, so are the $\boldsymbol{\mu}_j^{\boldsymbol{atac}}$ and $\boldsymbol{\sigma}_j^{\boldsymbol{atac}}$. Then, to obtain the $\mathbf{Z}_{\boldsymbol{\mu}}^{\boldsymbol{H}}$ and $\mathbf{Z}_{\boldsymbol{\sigma}}^{\boldsymbol{H}}$ in practice, two-layer graph convolutional networks (GCNs) is conducted on hybrid graph:

$$\mathbf{Z}_{\boldsymbol{\mu}}^{\boldsymbol{H}} = (GCN_{\mu}^{H}(\mathbf{X}^{\boldsymbol{H}},\mathbf{G}^{\boldsymbol{H}}))^T = (\widetilde{\mathbf{G}}^{\boldsymbol{H}} ReLU(\widetilde{\mathbf{G}}^{\boldsymbol{H}}(\mathbf{X}^{\boldsymbol{H}})^T\mathbf{W}_{\mathbf{0}}^{\boldsymbol{H}})\mathbf{W}_{\boldsymbol{\mu}}^{\boldsymbol{H}})^T,$$

$$\mathbf{Z}_{\boldsymbol{\sigma}}^{\boldsymbol{H}} = (GCN_{\sigma}^{H}(\mathbf{X}^{\boldsymbol{H}},\mathbf{G}^{\boldsymbol{H}}))^T = (\widetilde{\mathbf{G}}^{\boldsymbol{H}} ReLU(\widetilde{\mathbf{G}}^{\boldsymbol{H}}(\mathbf{X}^{\boldsymbol{H}})^T\mathbf{W}_{\mathbf{0}}^{\boldsymbol{H}})\mathbf{W}_{\boldsymbol{\sigma}}^{\boldsymbol{H}})^T$$

where $\mathbf{Z}_{\boldsymbol{\mu}}^{\boldsymbol{H}}, \mathbf{Z}_{\boldsymbol{\sigma}}^{\boldsymbol{H}} \in \mathbb{R}^{k\times(n_1+|\mathbb{A}|)}$ share the same first-layer parameter matrix of $\mathbf{W}_{\mathbf{0}}^{\boldsymbol{H}} \in \mathbb{R}^{g\times d}$ but different second-layer parameter matrices of $\mathbf{W}_{\boldsymbol{\mu}}^{\boldsymbol{H}} \in \mathbb{R}^{d\times k}$ and $\mathbf{W}_{\boldsymbol{\sigma}}^{\boldsymbol{H}} \in \mathbb{R}^{d\times k}$; $ReLU(\cdot) = max(0, \cdot)$ is the activation function; $\widetilde{\mathbf{G}}^{\boldsymbol{H}} = \mathbf{D}^{-\frac{1}{2}}\mathbf{G}^{\boldsymbol{H}}\mathbf{D}^{-\frac{1}{2}}$ is the symmetrically normalized adjacency matrix aiming to effective training, where $\mathbf{D}$ is the diagonal degree matrix of $\mathbf{G}^{\boldsymbol{H}}$, respectively. Similarly, $\mathbf{Z}_{\boldsymbol{\mu}}^{\boldsymbol{atac}}$, $\mathbf{Z}_{\boldsymbol{\sigma}}^{\boldsymbol{atac}} \in \mathbb{R}^{k\times n_2}$ can be calculated in the same way. Then, the final cell embeddings of the hybrid graph and the ATAC graph can be obtained from:

$$\mathbf{Z}^{\boldsymbol{H}} = \mathbf{N}^{\boldsymbol{H}} \odot \mathbf{Z}_{\boldsymbol{\sigma}}^{\boldsymbol{H}} + \mathbf{Z}_{\boldsymbol{\mu}}^{\boldsymbol{H}},$$

$$\mathbf{Z}^{\boldsymbol{atac}} = \mathbf{N}^{\boldsymbol{atac}} \odot \mathbf{Z}_{\boldsymbol{\sigma}}^{\boldsymbol{atac}} + \mathbf{Z}_{\boldsymbol{\mu}}^{\boldsymbol{atac}}$$

where $\mathbf{N}^{\boldsymbol{H}} \in \mathbb{R}^{k\times(n_1+|\mathbb{A}|)}$ and $\mathbf{N}^{\boldsymbol{atac}} \in \mathbb{R}^{k\times n_2}$ are random matrices generated form standard normal distributions; $\mathbf{A} \odot \mathbf{B}$ represents the Hadamard product of two matrices. Finally, the loss function which regulates the latent variable $\mathbf{Z}^{\boldsymbol{H}}$ and $\mathbf{Z}^{\boldsymbol{atac}}$ from Gaussian distribution is optimized as:

$$\mathcal{L}_1 = -(KL\left(q\left(\mathbf{Z}^{\boldsymbol{H}}\middle|\mathbf{X}^{\boldsymbol{H}},\mathbf{G}^{\boldsymbol{H}}\right)\middle|,p\left(\mathbf{Z}^{\boldsymbol{H}}\right)\right) + KL\left(q\left(\mathbf{Z}^{\boldsymbol{atac}}\middle|\widetilde{\mathbf{X}}^{\boldsymbol{atac}},\mathbf{G}^{\boldsymbol{atac}}\right)\middle|,p\left(\mathbf{Z}^{\boldsymbol{atac}}\right)\right))$$

where $KL\big(q(\cdot)\big|,p(\cdot)\big)$ is the Kullback-Leibler (KL) divergence between distribution $q(\cdot)$ and $p(\cdot)$; As Gaussian priors, $p\left(\mathbf{Z}^{\boldsymbol{H}}\right) = \prod_i \mathcal{N}(\boldsymbol{z_i}|0,\boldsymbol{I})$ and $p(\mathbf{Z}^{\boldsymbol{atac}}) = \prod_j \mathcal{N}\left(\boldsymbol{z_j}\middle|0,\boldsymbol{I}\right)$. According to the solution B in VAE original paper[3], since both prior $p(\mathrm{Z}^{H}) = \prod_i \mathcal{N}(\boldsymbol{z_i}|0,\boldsymbol{I})$ and posterior approximation $q\left(\mathbf{Z}^{\boldsymbol{H}}\middle|\mathbf{X}^{\boldsymbol{H}},\mathbf{G}^{\boldsymbol{H}}\right)$ are Gaussian, $KL\left(q\left(\mathbf{Z}^{\boldsymbol{H}}\middle|\mathbf{X}^{\boldsymbol{H}},\mathbf{G}^{\boldsymbol{H}}\right)\middle|,p\left(\mathbf{Z}^{\boldsymbol{H}}\right)\right)$ can be computed as:

$$KL\left(q\left(\mathbf{Z}^{\boldsymbol{H}}\middle|\mathbf{X}^{\boldsymbol{H}},\boldsymbol{G}^{\boldsymbol{H}}\right)\middle|,p\left(\mathbf{Z}^{\boldsymbol{H}}\right)\right) = -\frac{1}{2}\sum_{d=1}^{k}\sum_{i=1}^{n_1+|\mathbb{A}|}(1 + 2log(\sigma_{d,i}^{H}) - (\mu_{d,i}^{H})^2 - (\sigma_{d,i}^{H})^2)$$

where k is the dimension number of the embedding space, $\sigma_{d,i}^{H}$ and $\mu_{d,i}^{H}$ are the element in d-th row and i-th column of $\mathbf{Z}_{\boldsymbol{\mu}}^{\boldsymbol{H}}$ and $\mathbf{Z}_{\boldsymbol{\sigma}}^{\boldsymbol{H}}$, respectively.

Similarly,

$$KL\left(q\left(\mathbf{Z}^{\boldsymbol{atac}}\middle|\widetilde{\mathbf{X}}^{\boldsymbol{atac}},\mathbf{G}^{\boldsymbol{atac}}\right)\middle|,p\left(\mathbf{Z}^{\boldsymbol{atac}}\right)\right) = -\frac{1}{2}\sum_{d=1}^{k}\sum_{j=1}^{n_2}(1 + 2log(\sigma_{d,j}^{atac}) - (\mu_{d,j}^{atac})^2 - (\sigma_{d,j}^{atac})^2)$$

where k is the dimension number of the embedding space, $\sigma_{d,j}^{atac}$ and $\mu_{d,j}^{atac}$ are the element in d-th row and i-th column of $\mathbf{Z}_{\boldsymbol{\mu}}^{\boldsymbol{atac}}$ and $\mathbf{Z}_{\boldsymbol{\sigma}}^{\boldsymbol{atac}}$, respectively. Thus, loss $\mathcal{L}_1$ can be rewritten as:

$$\mathcal{L}_1 = -\frac{1}{2}\sum_{d=1}^{k}(\sum_{i=1}^{n_1+|\mathbb{A}|}(1 + 2log(\sigma_{d,i}^{H}) - (\mu_{d,i}^{H})^2 - (\sigma_{d,i}^{H})^2) + \sum_{j=1}^{n_2}(1 + 2log(\sigma_{d,j}^{atac}) - (\mu_{d,j}^{atac})^2 - (\sigma_{d,j}^{atac})^2))$$

where k is the dimension number of the embedding space, $\sigma_{d,i}^{H}$ ($\sigma_{d,j}^{atac}$) and $\mu_{d,i}^{H}$ ($\mu_{d,j}^{atac}$) are the element in d-th row and i-th (j-th) column of $\mathbf{Z}_{\boldsymbol{\mu}}^{\boldsymbol{H}}$ ($\mathbf{Z}_{\boldsymbol{\mu}}^{\boldsymbol{atac}}$) and $\mathbf{Z}_{\boldsymbol{\sigma}}^{\boldsymbol{H}}$ ($\mathbf{Z}_{\boldsymbol{\sigma}}^{\boldsymbol{atac}}$), respectively.

Supplementary Note 4: Differences between the initial and reconstructed RNA-ATAC graph

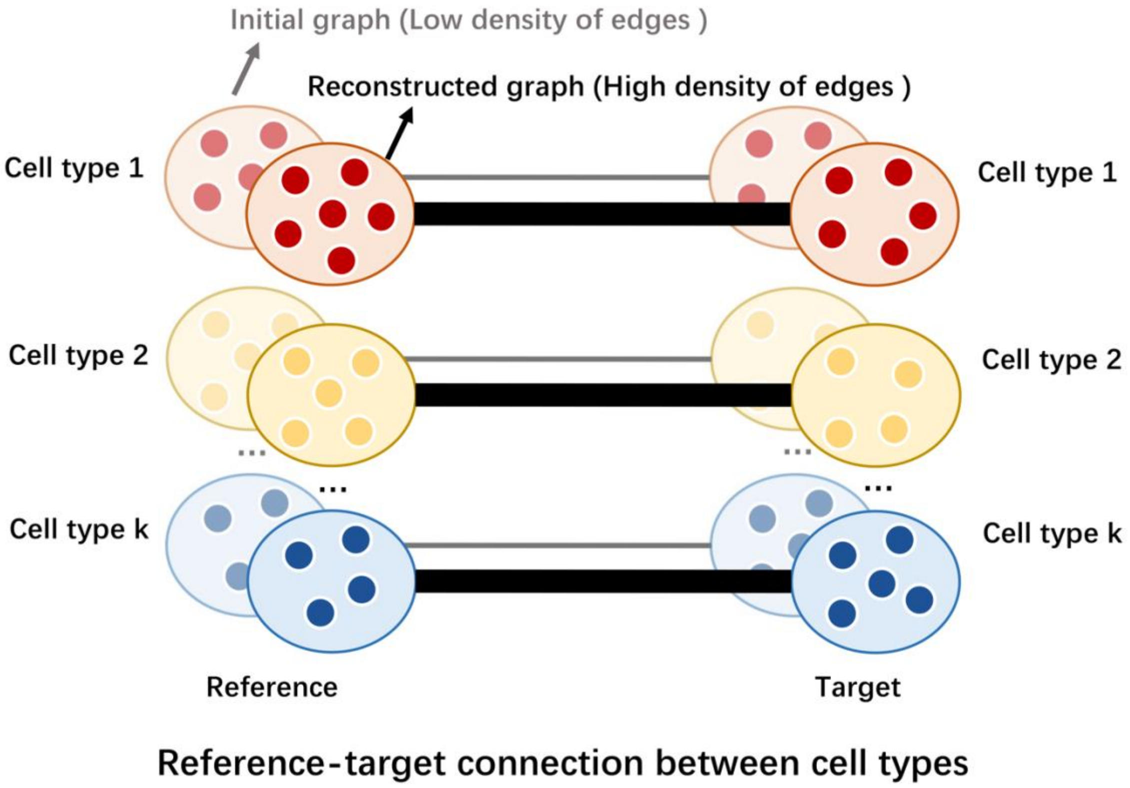

Firstly, we elaborated on the role of the reconstructed graph in our model. Since our model is based on the Variational Graph Autoencoder (VGAE)[2] which is the graph version of the Variational Autoencoder (VAE) model[3], it is crucial to understand the workings of VAE. The VAE model embeds the original data space into a latent space and assumes that different categories are sampled from a multivariate Gaussian distribution. It then uses the latent space to reconstruct the original space and minimizes the difference between the initial one and the reconstructed one. This ensures that the trained latent space contains most of the information of the original space. Following a similar approach, VGAE takes the initial graph as input, projects it into the latent space, and reconstructs a new graph. According to previous research[4], the reconstructed graph produced by VGAE is typically used to perform link prediction tasks. This is because, compared to the initial graph, the reconstructed graph possesses a higher density of edges, therefore being more informative. In the context of HyGAnno, this rich, detailed information allows for more accurate and robust cell annotation. In our research, the

edges of the reconstructed RNA-ATAC graph G are supposed to be highly linked between the cells in reference and target. To evidence the reconstructed graph has a higher Density of Linked Edges (DLE) value than that of the initial one, we calculated the DLE value between cell type i in reference and cell type j in target by the following metric:

$$DLE_{i,j} = \frac{\sum \widehat{\mathrm{G}}_{c_i^{ref}, c_j^{tar}}}{\mathrm{N}_i^{ref} \mathrm{N}_j^{tar}}$$

where N_i^{ref} and N_j^{tar} are the size of cell type i in reference and cell type j in target, respectively; $\widehat{\mathrm{G}}_{c_i^{ref}, c_j^{tar}}$ is the edge weight between two cells with the cell type i of cell and j. The DLE values of the initial and reconstructed graph are calculated and visualized to demonstrate the higher density of linked edges in reconstructed graph (Supplementary Fig. 6).

Supplementary Note 5: Details of data preparation for benchmarking

Datasets for RNA-referenced methods. We downloaded the processed SNARE-seq data of the mouse brain from the NCBI GEO (GSE126074) and analyzed cell clusters using Seurat. This provided 14 cell clusters, and we annotated the clusters based on the marker gene expression and functions mentioned in the original publication[5]. To prepare the chromatin accessibility matrix, we remained the ATAC-seq peaks included in larger than 10 cells in the SNARE-seq data. To create the gene activity matrix, we run *GeneActivity* function in Signac to estimate reads mapped within the 2-Kb upstream and gene-body regions. The intersection of highly variable genes from the gene expression matrix and gene activity matrix are used as features. As a result, we created the gene expression matrix ($\mathbf{X}^{rna}$) of 1,305-genes-by-8,055-cells, the chromatin accessibility matrix ($\mathbf{X}^{atac}$) of 64,064-peaks-by-8,055-cells, and the gene activity matrix ($\mathbf{X}^{gam}$) of 1,305-genes-by-8,055-cells. For the mouse lung dataset, the scRNA-seq and scATAC-seq data are downloaded from the database of *Tabula Muris*[6] and the atlas of the adult mice chromatin accessibility[7], respectively. The gene activity matrix calculated by Cicero[8] is obtained directly from the data website same as the scATAC-seq data. As a result, we prepared $\mathbf{X}^{rna}$ of 1,822-genes-by-2,623-cells, $\mathbf{X}^{atac}$ of 51,465-peaks-by-7,499-cells, and $\mathbf{X}^{gam}$ of 1,822-genes-by-7,499-cells with 8 cell types. For human peripheral blood mononuclear cells (PBMC) datasets, we processed the scRNA-seq and scATAC-seq data with 10 cell types from GSE139369 based on a previous research[9] and obtained $\mathbf{X}^{rna}$ of 1,825-genes-by-13,345-cells, $\mathbf{X}^{atac}$ of 24,322-peaks-by-7,828-cells, and $\mathbf{X}^{gam}$ of 1,825-genes-by-7,828-cells. The gene activity matrix calculated by Cicero is downloaded together with the scATAC-seq data. For bone marrow mononuclear cells (BMMC) datasets, we downloaded the raw data from the same repository as PBMC and finally obtained $\mathbf{X}^{rna}$ of 1,788-genes-by-11,884-cells, $\mathbf{X}^{atac}$ of 21,201-peaks-by-14,753-cells, and $\mathbf{X}^{gam}$ of 1,788-genes-by-14,753-cells with 13 cell types.

Datasets for ATAC-referenced methods. We used the scATAC-seq PBMC datasets from three healthy donors[9,10]: D10, D12, and D_{rep1}, which are 452,004-peaks-by-2,588-cells with 9 cell types, 452,004-peaks-by-3,070-cells with 9 cell types, and 127,541-peaks-by-9,060-cells with 6 cell types, respectively. Among them, D10 and D12 are downloaded from GSE139369, D_{rep1}

is downloaded from GSE129785. For convenience, we renamed the peak matrices of D10, and D12, and D_{rep1} as peak matrices 1, 2, and 3, respectively. As Cellcano requires gene-level matrices as inputs, the gene activity matrix of each peak matrix is necessary. For peak matrices 1 and 2, the corresponding gene activity matrices calculated by Cicero are already provided in the original research. The gene activity matrix of peak matrix 3 is unavailable, prompting us to generate it ourselves using the pipeline recommended by Cicero. Consequently, the gene activity matrices of peak matrices 1, 2, and 3 are named as gene activity matrices 1, 2, and 3, respectively. On the other hand, EpiAnno uses peak-level matrices as inputs, and it specifically requires that the peak regions in the reference and target are identical. To satisfy this requirement, we regenerate the target peak matrix through the *FeatureMatrix* function in the Signac package based on the peaks of the reference data and the fragment file of the target data acquired from the original work.

Supplementary Note 6: Details of data preparation for tumor cell detection

The scRNA-seq data used in the tumor cell detection task was obtained from a previous study[11]. In this work, the authors analyzed 26 primary tumors with three broad subtypes, including luminal, HER2+, and triple negative breast cancer (TNBC). After the initial quality control steps, we ended up with a dataset consisting of 75,443 single cells. Because the neoplastic cells and normal epithelial cells often exhibit similar gene expression patterns, it can be challenging to annotate them based only on gene markers. To address this, we employed a method called scATOMIC, a pan-cancer classifier that has been trained on more than 300,000 cells[12], to estimate single-cell copy number variant (CNV) profiles. With the help of scATOMIC, we identified seven cell types, including tumor cells. To accelerate the training of HyGAnno, we used a subset of the original scRNA-seq data, resulting $\mathbf{X}^{rna}$ of 1,539-genes-by-15,088-cells, with 6 healthy cell types and 1 tumor cell type.

For scATAC-seq data, we selected a dataset derived from16 patients with three broad cancer subtypes, consistent with the scRNA-seq mentioned above. Due to the inaccessibility of the peak matrix and cell annotation information in the original study, we decided to process the raw data ourselves. First, we merged fragment files derived from 16 patients. Then, *CallPeaks* function and *FeatureMatrix* function in the Signac package are employed to enrich reads to peaks and generate peak matrix, respectively. While scRNA-seq data allowed for the usage of CNV profiles to differentiate between tumor cells and normal epithelial cells, there is no such direct method exists for scATAC-seq. This is due to scATAC-seq data quantifying the open accessibility level of chromatin regions, rather than gene expression like scRNA-seq. Hence, instead of high-resolution annotation, we transferred the peak matrix to the gene activity matrix by *GeneActivity* function of Signac package and roughly annotated cells by marker genes of normal cells, identifying six cell types except the tumor cell. Finally, $\mathbf{X}^{atac}$ of 65,526-peaks-by-11,116-cells, and $\mathbf{X}^{gam}$ of 1,539-genes-by-11,116-cells with 6 healthy cell types are obtained.

Supplementary Note 7: Cell embedding generation methods

Given the sparse and high-dimensional peak matrix in scATAC-seq data, directly exploring cell similarity in the original cell space is challenging. However, cell embedding space, a low-dimensional representation of the original space, can assist researchers in investigating the chromatin accessibility differences between the cell types. There are various ways to generate the cell embedding space, here we mainly showed the comparison methods used in this paper. For cell embeddings with the latent semantic indexing (LSI) method, we used the functions available in Signac[1]. First, term frequency-inverse document frequency (TF-IDF) normalization is performed on the raw peak matrix using *RunTFIDF* function. The TF-IDF normalized peak matrix is then subjected to singular value decomposition (SVD) via the *RunSVD* function. Following the instructions of Signac, we discarded the first SVD component, as it typically captures technical variation rather than biological variation. We then used the SVD components from top 2 to 29 to compute the ASW scores.

For cell embeddings with PCA, we followed the pipeline suggested by Seurat[13] and applied PCA to the gene activity matrix with 2000 highly variable genes. The first 30 PCs are used to compute the ASW scores.

For cell embeddings with scJoint[14] and scGCN[15], we executed the scripts provided by the authors with default parameters. Since these methods employ deep neural networks, the output layers with 64 and 32 dimensions are treated as cell embeddings for scJoint and scGCN, respectively. Both methods automatically output cell embedding files, which can be used to compute the ASW scores.

For our method, HyGAnno, similar to scJoint and scGCN, we used the output layer as the cell embedding to calculate the ASW scores. Different from the scGCN and scJoint, we set the dimension number of the output layer equal to the cell type number of reference data.

Supplementary Note 8: Evaluation metrics

Evaluation of cell annotation performance. Assuming that the ground truth of target data and the predicted cell label list by different methods are $\boldsymbol{x}$ and $\boldsymbol{y}$, respectively. For the general evaluation of the cell annotation performance, we used Accuracy (ACC) and Normalized Mutual Information (NMI):

$$ACC(X,Y) = \frac{Correct\ cell\ numbers}{All\ cell\ numbers} \in [0,1]$$

$$NMI = \frac{MI(\boldsymbol{x},\boldsymbol{y})}{\sqrt{H(\boldsymbol{x}) \cdot H(\boldsymbol{y})}} \in [0,1]$$

where $MI(\cdot,\cdot)$ is the mutual entropy between two list; $H(\cdot)$ is the entropy of a list. In this NMI formulation, we took the geometric mean to normalize the mutual information.

We also used F1 score to elaborate the prediction performance in cell type i:

$$F1_i = 2 \cdot \frac{Precision_i \cdot Recall_i}{Precision_i + Recall_i} \in [0,1]$$

where $Precision_i = \frac{TP_i}{TP_i+FP_i}$; $Recall_i = \frac{TP_i}{TP_i+FN_i}$; TP_i, FP_i, and FN_i are the number of true positive cells, false positive cells, and false negative cells of cell type i, respectively. Also, we have the weighted F1 score normalized by the size of different cell types:

$$Weighted\ F1\ score = \frac{\sum_{i=1}^{k} F1_i \cdot c_i}{\sum_i^k c_i} \in [0,1]$$

where $F1_i$ and c_i are the F1 score and the cell number of cell type i, respectively, k is the number of cell types. Similarly, we have the average F1 score:

$$Avg.F1\ score = \frac{\sum_{i=1}^{k} F1_i}{k} \in [0,1]$$

Evaluation of cell embedding performance. The Silhouette Width (SW)[16] is a metric that quantifies the quality of clustering assignments. For a given cell, it calculates the average distance to other cells in its own cluster (inner-cluster distance) and compares this to the average distance to cells in the other cluster (inter-cluster distance). We calculated the SW score for each cell type i as:

$$SW_i = \frac{b-a}{max(a,b)} \in [-1,1]$$

where a is the inner-cluster distance and b is the inter-cluster distance. $ASW = \frac{1}{k}\sum_{i=1}^{k} SW_i$ is the average SW among all cell types, where k is the number of cell types. Higher ASW means

well-separate cell clusters, that cells with the same labels are close with each other; cells with distinct labels are far away from each other.

Supplementary Note 9: Downstream analysis

TF motif enrichment. The chromVAR R package[17] version 1.18.0 was used to calculate the motif enrichment of TFs in mouse lung data. UCSC version mm9 was used as the genome reference to detect motifs, which were then used as the input for JASPAR2022, to map these motifs to TFs under experimental validation.

Detection of cell type-specific peaks. To elaborate on the relationship between peak-level and latent features, we simplified our model into one-layer neural network. After the model had been fully trained (ACC=0.90, NMI=0.80), we extracted the hidden parameter matrix from the ATAC channel. For each cell type-specific latent feature, we selected the top 500 peaks according to their feature values as the cell type-specific peaks, where an overlapping threshold of 200 bases is applied to map promoter-distal peaks to cell type-specific enhancers from the scEnhancer database.

Trajectory inference on cell embedding space. The destiny R package[18] version 3.10.0 was used to infer the cell trajectories. We applied the inner functions using cell embedding as the input and calculated the diffusion pseudotime with all default parameters. The first two diffusion components were used for trajectory visualization.

Copy number variation (CNV) calculation for scATAC-seq data. We inferred the CNV signals by running the scripts provided in a previous work[10]. After merging the raw scATAC-seq fragment files from different patients, we separated the chromosomes into 10-Mb windows and returned the average log2(fold-change) of fragment count falling into each window against the 100 nearest neighbors, based on GC content. Regions with a higher log2(fold-change) were considered candidates for amplification. To clearly illustrate the CNV pattern between tumor cells and normal cells in breast cancer, we subtracted the mean value of each window from the CNV scores of TME cells and obtained the normalized CNV scores[19].

Supplementary Figures

Supplementary Figure 1

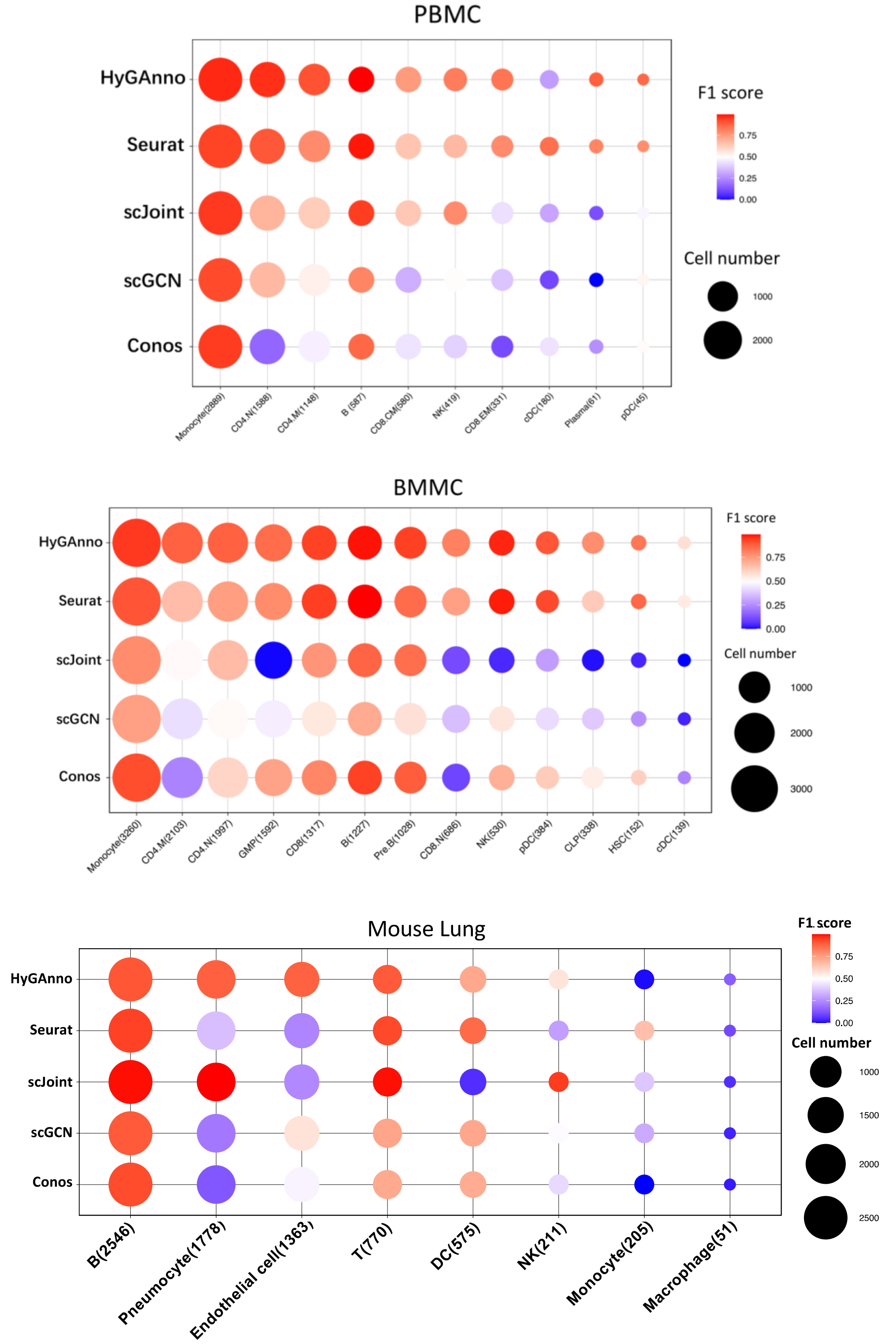

Supplementary Fig. 1. *F1 score regarding each cell type of HyGAnno with Seurat, scJoint, scGCN, and Conos among PBMC, BMMC, and mouse lung datasets. The number in bracket is the cell number of each cell type.*

Supplementary Figure 2

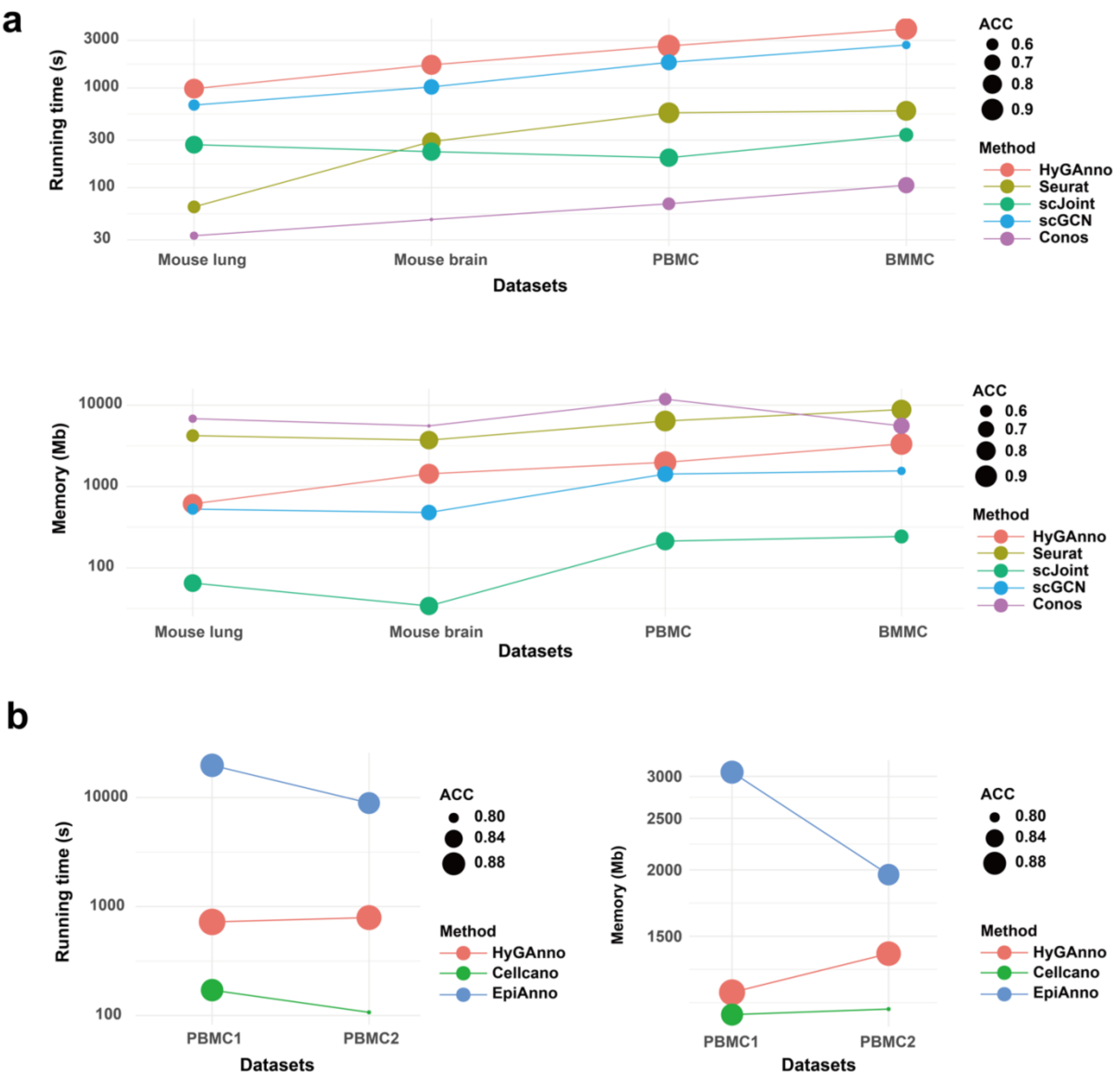

Supplementary Fig. 2. *Computational efficiency comparison. Running time and memory requirement of HyGAnno compared with* ***(a)*** *RNA-referenced methods including Seurat, scJoint, scGCN, and Conos on mouse lung, mouse brain, PBMC, and BMMC datasets; and* ***(b)*** *ATAC-referenced methods including EpiAnno and Cellcano on two PBMC datasets. The x-axis is ordered by the number of cells in the target datasets. The size of points indicates the value of prediction accuracy.*

Supplementary Figure 3

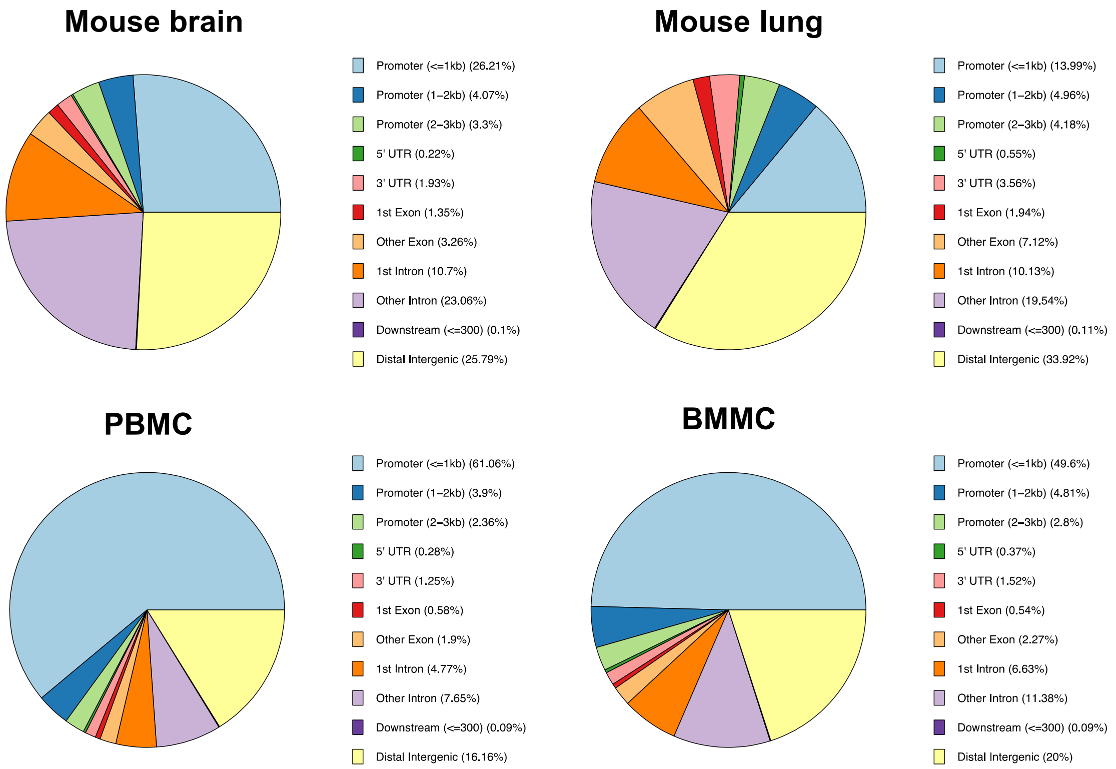

Supplementary Fig. 3. *Peak annotation for all genome-wide accessible peaks of mouse brain, mouse lung, PBMC, and BMMC used in HyGAnno.*

Supplementary Figure 4

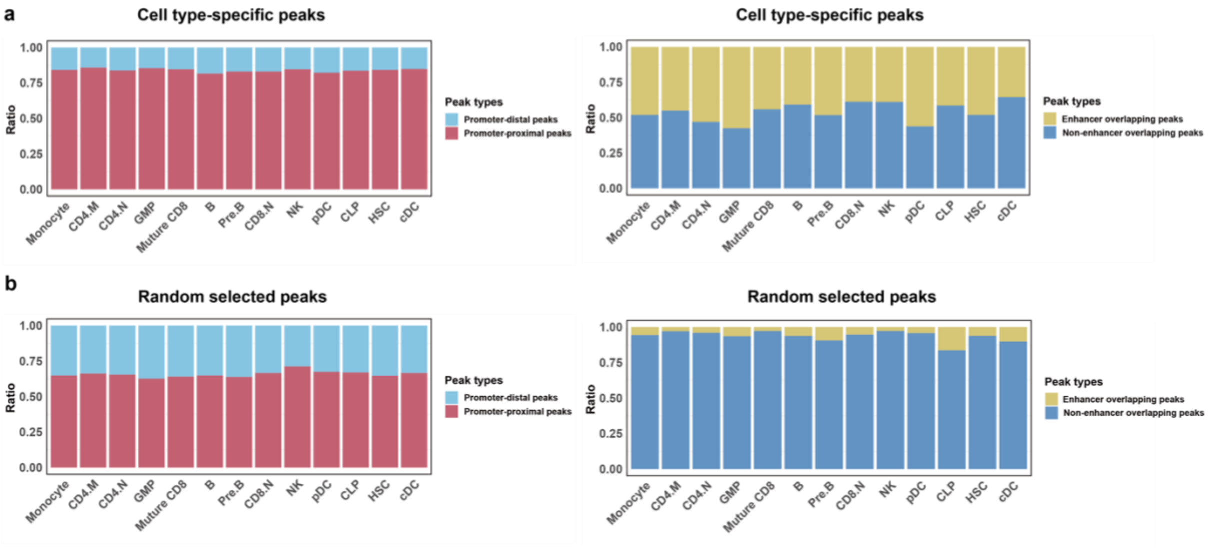

Supplementary Fig. 4. (a) *Peak annotation based on the cell type-specific peaks of the BMMC dataset detected by HyGAnno.* ***(b)*** *Peak annotation based on randomly selected peaks. Left: Distance-based peak annotation; Right: Enhancer-based peak annotation.*

Supplementary Figure 5

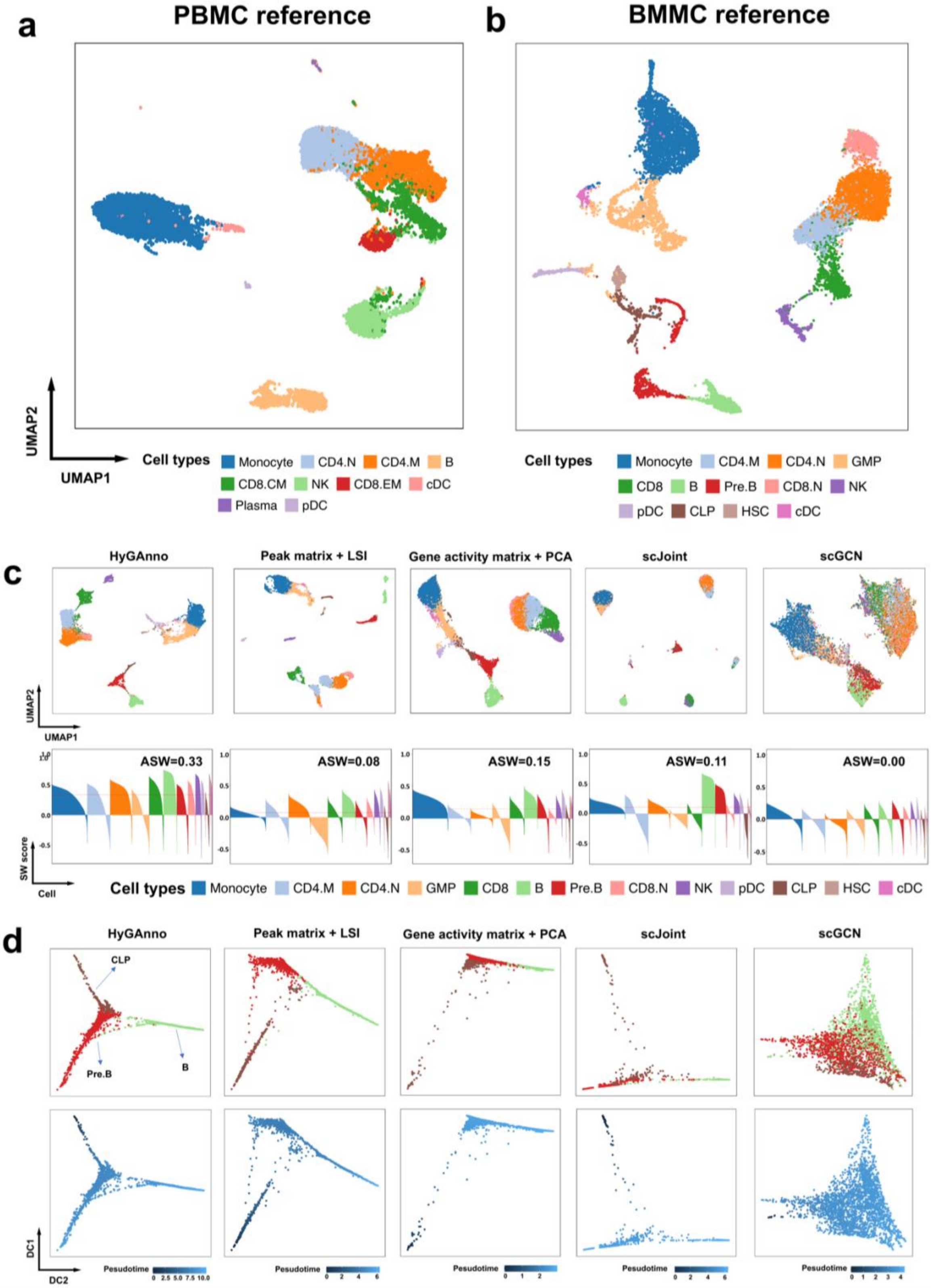

Supplementary Fig. 5. (a, b) *UMAP plots of cell embeddings in scRNA-seq reference data of PBMC and BMMC, respectively. Cell embeddings are obtained by applying PCA to the gene*

expression matrix. ***(c)*** *UMAP plots (upper row) and the corresponding SW scores (bottom row) of BMMC cell embeddings. The cells are colored by ground truth cell types. Four T cell subtypes are surrounded by red circles.* ***(d)*** *Trajectory analysis based on the cell embeddings of CLP cells, Pre. B cells, and B cells. The cells are colored by ground truth cell types (upper row) and pseudotime calculated by DPT (bottom row).*

Supplementary Figure 6

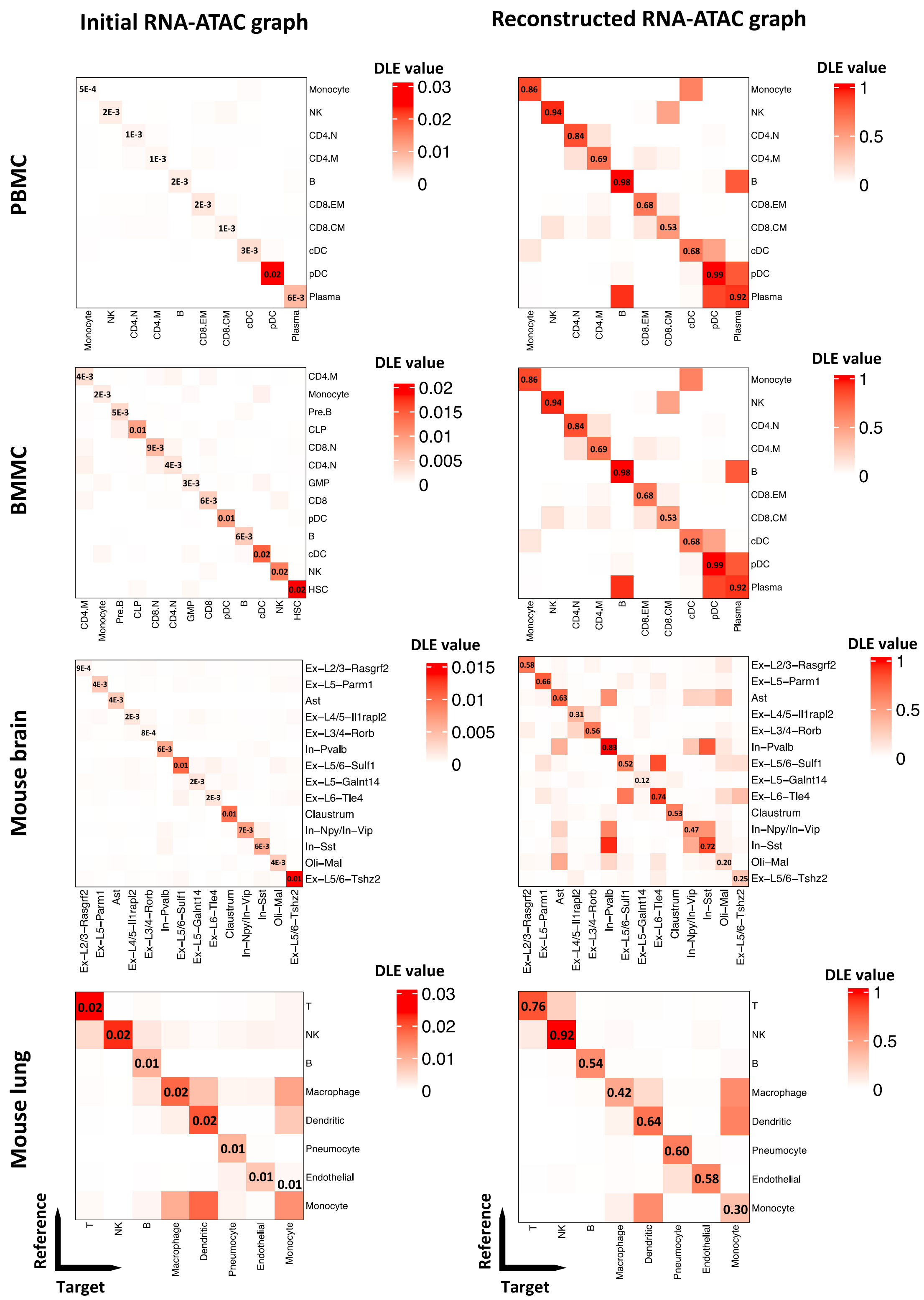

Supplementary Fig. 6. *Reconstructed RNA-ATAC graphs show higher density of edges compared with the initial one. Rows are the cells in reference data; columns are the cells in target data.*

Supplementary Figure 7

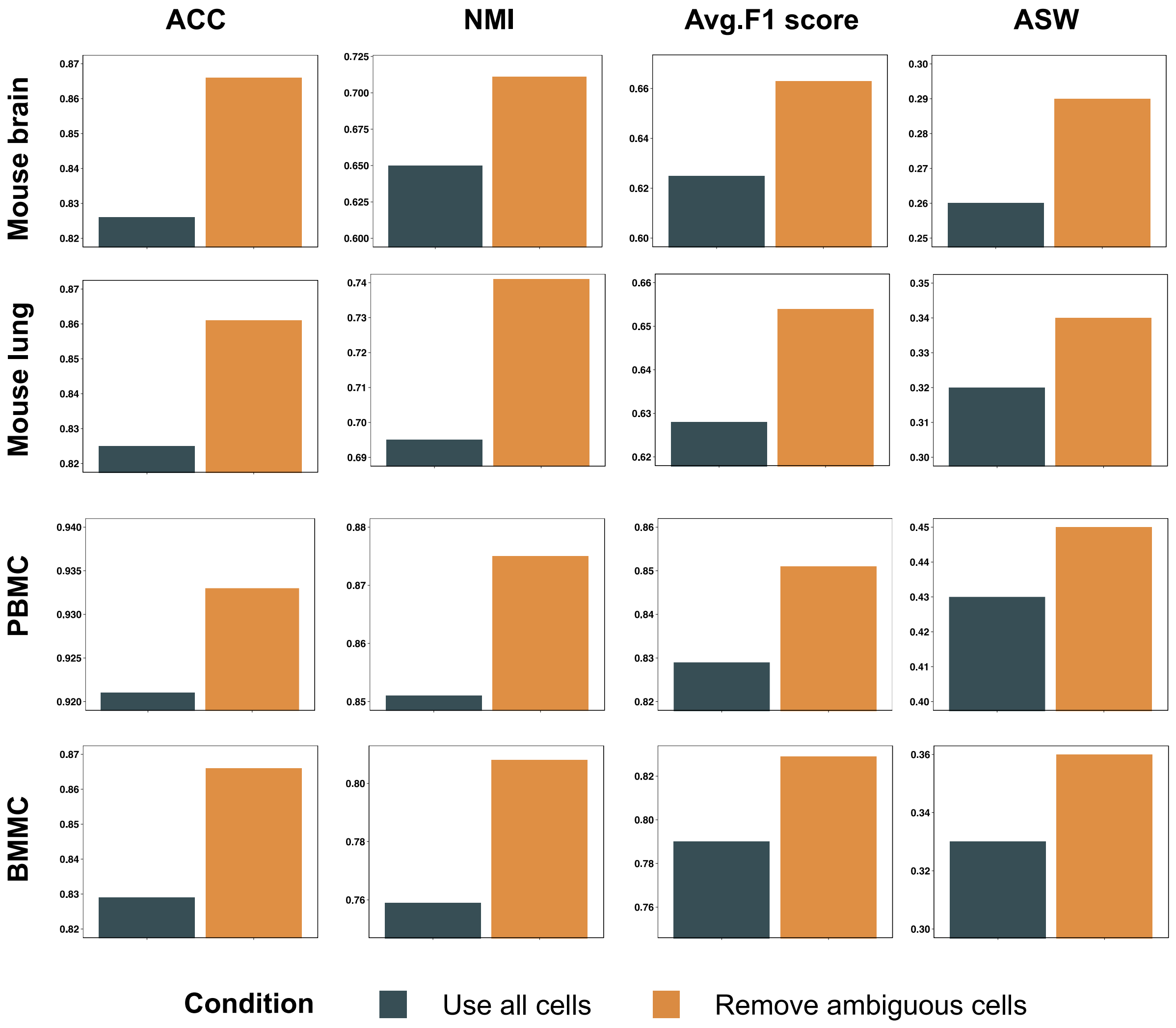

Supplementary Fig. 7. *Cell annotation performance comparisons with and without the ambiguous cells by ACC, NMI, average F1 score, and ASW.*

Supplementary Figure 8

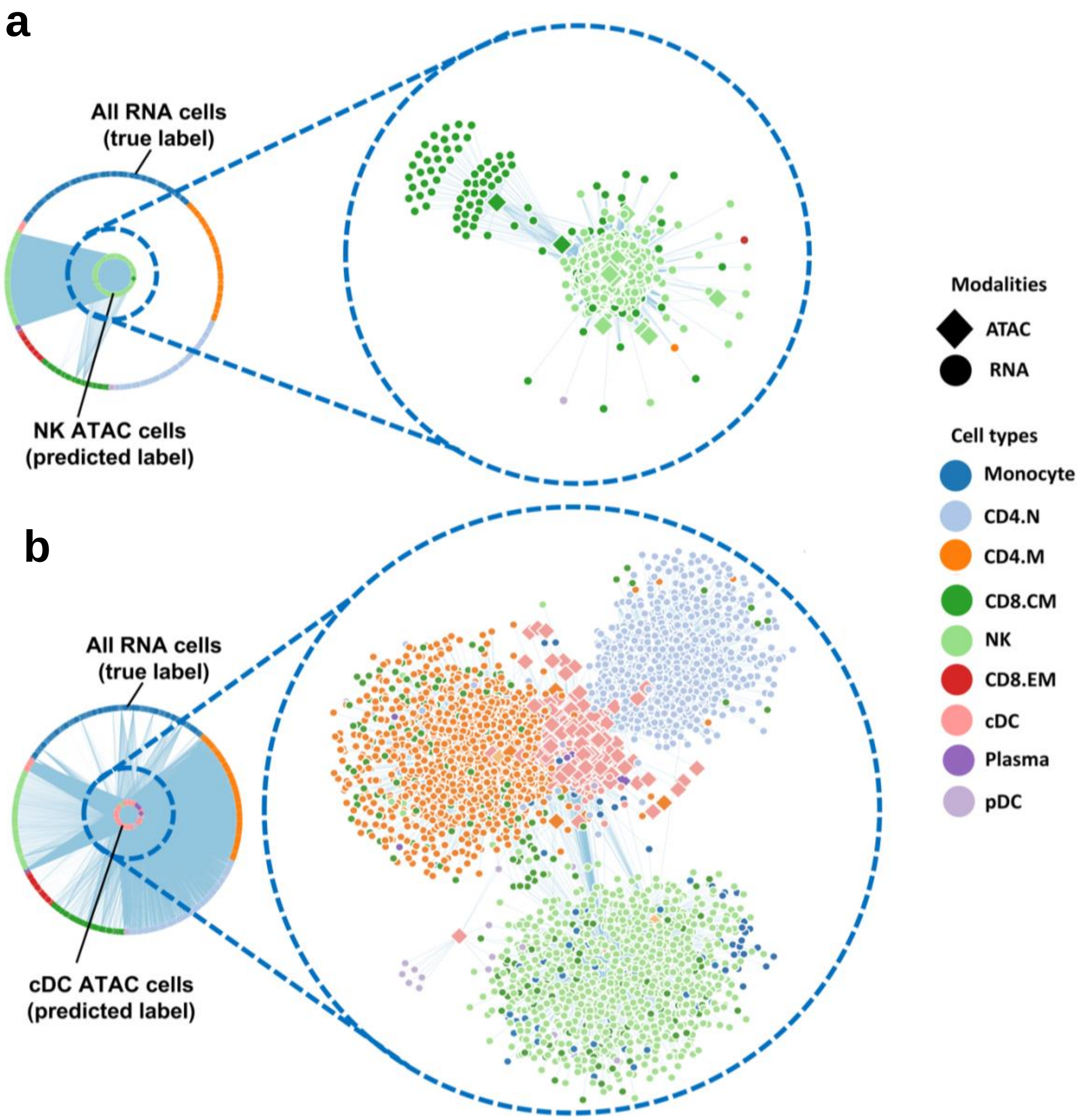

Supplementary Fig. 8. *(**a**) The reconstructed graph between confident cells and RNA clusters in PBMC data which are colored by predicted and true cell types, respectively. (**b**) The connectivity pattern between ambiguous cells and RNA clusters in PBMC data are colored by predicted and true cell types, respectively.*

Supplementary Figure 9

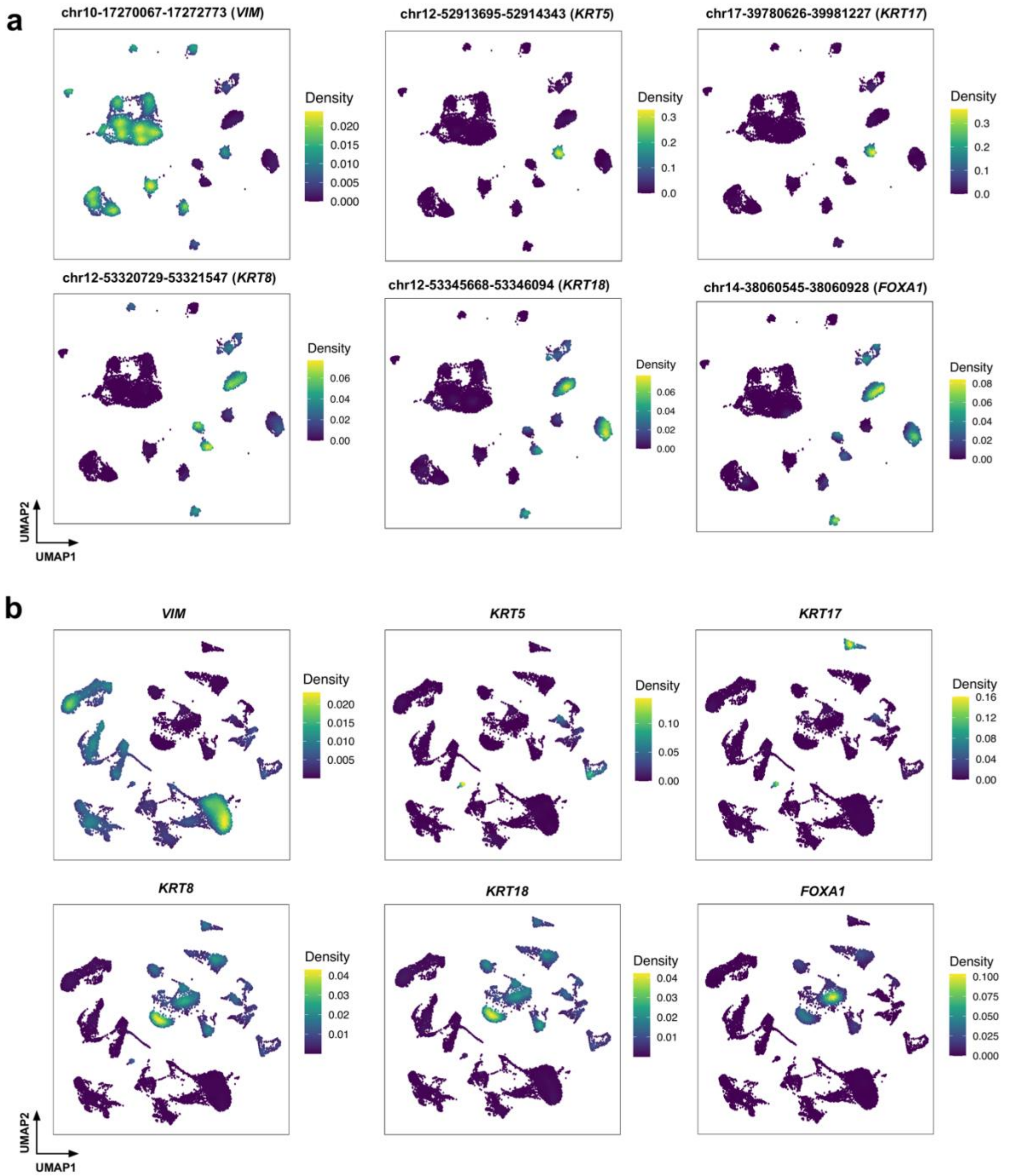

Supplementary Fig. 9. *(**a**) The accessibility level of peaks in the promotor regions of significant genes in scATAC-seq data. (**b**) The expression level of significant genes in scRNA-seq data.*

Supplementary Figure 10

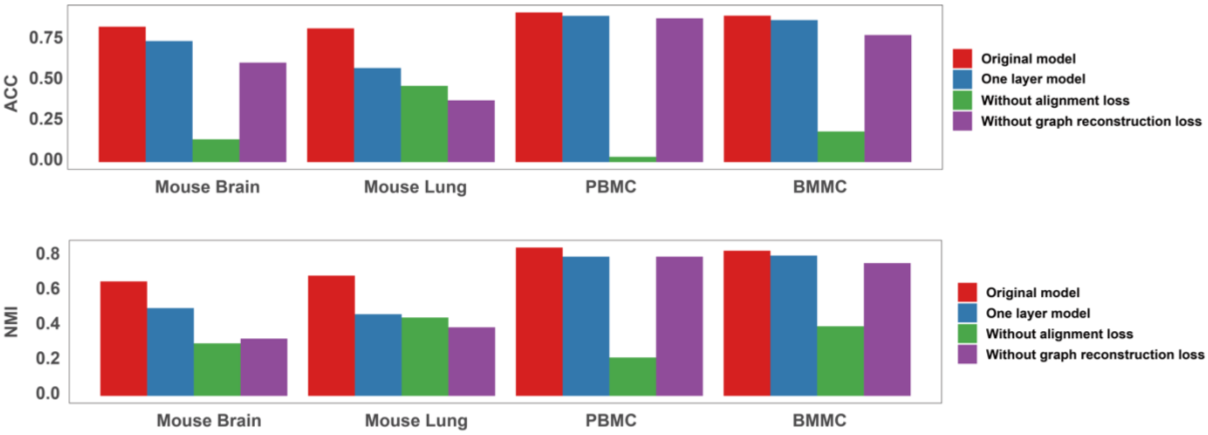

Supplementary Fig. 10. *In addition to the original model, three modified models were tested: one with a single layer, one excluding the alignment loss function (Eq. 6), and another without the graph reconstruction loss function (Eq. 7). These modifications were employed to evaluate their individual contributions to the final prediction performance (ACC and NMI) across four datasets: mouse brain, mouse lung, PBMC, and BMMC.*

Supplementary Figure 11

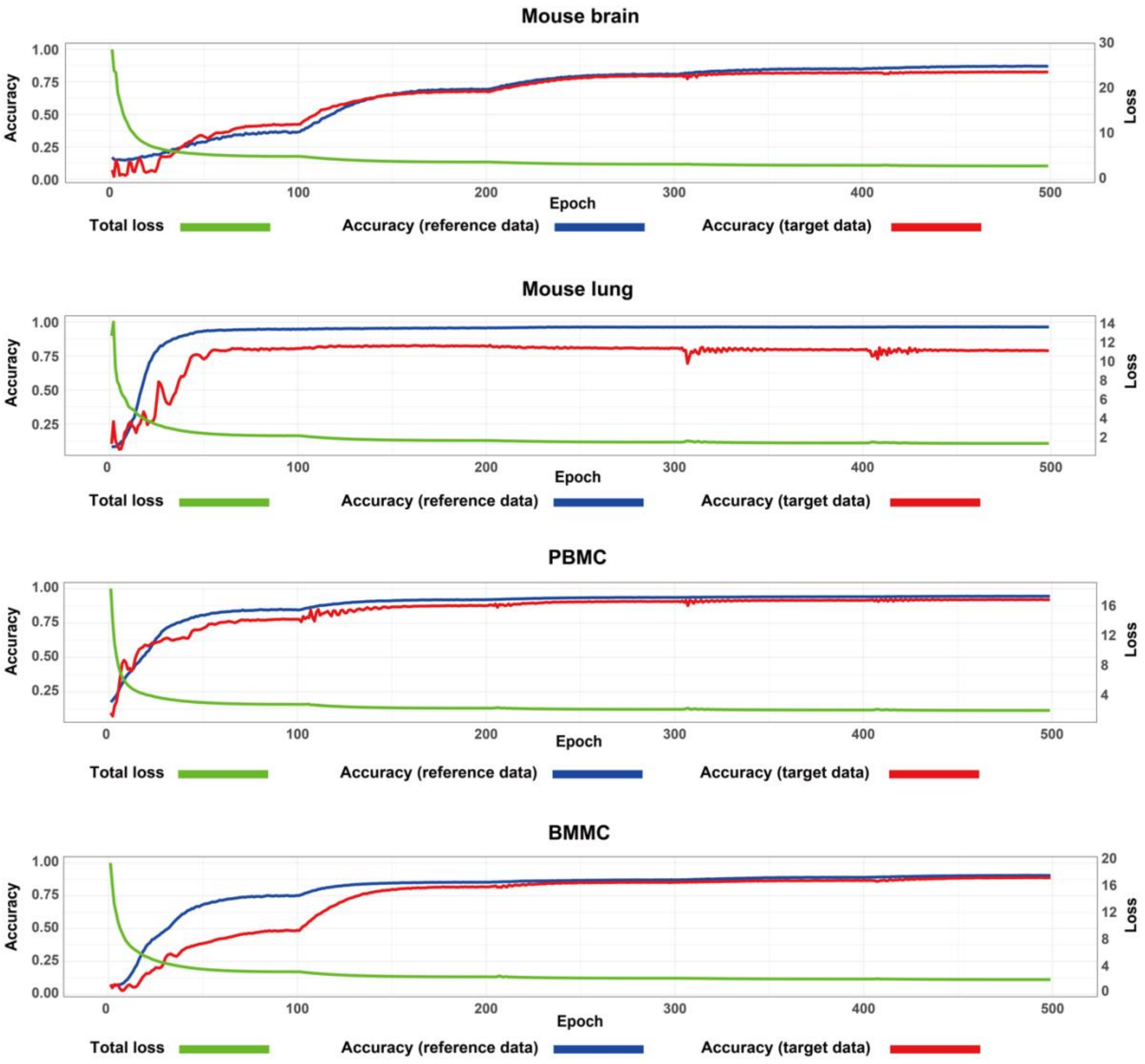

Supplementary Fig. 11. *The epoch vs. Accuracy (left y axis) and Total loss (right y axis) curves among four datasets, which are mouse brain, mouse lung, PBMC, and BMMC. The dynamic learning rate strategy, 'CosineAnnealingWarmRestarts' from Pytorch, was utilized. The learning rate, initially set at 0.0001, decreases with each epoch and automatically resets to the initial value every 100 epochs. The plots illustrate the results in 500 epochs.*

Supplementary Figure 12

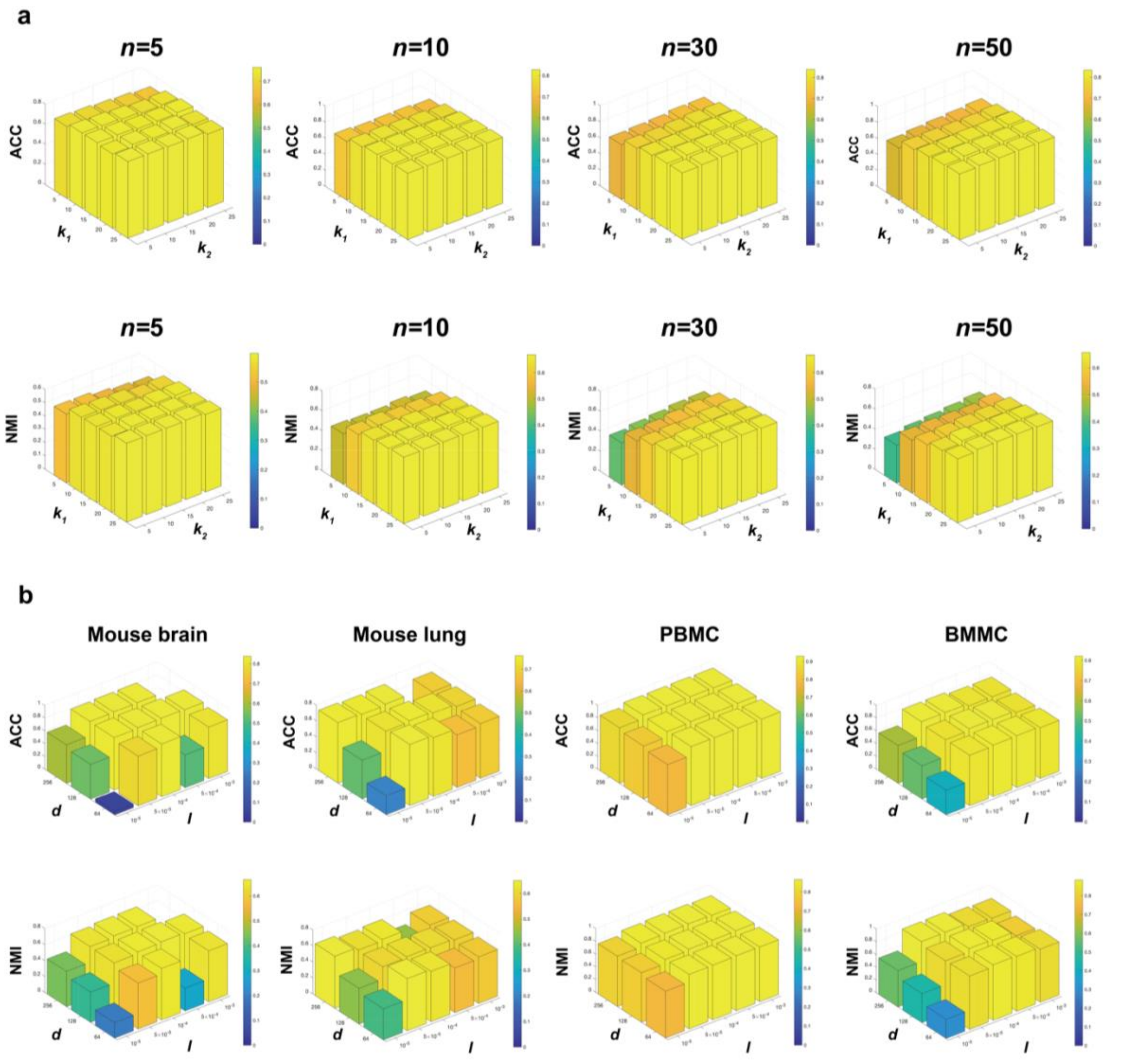

Supplementary Fig. 12: (a) *Performance of HyGAnno on mouse brain dataset based on different parameter settings of graph construction part. The dimension number* n *of low-dimension space is set from 5 to 50, while the shared* k*-nearest neighbors* k_1 *and* k_2 *for constructing graph and detecting anchor cells, respectively, are both set from 5 to 25. Accuracy (ACC) and Normalized Mutual Information (NMI) are evaluated.* ***(b)*** *Performance of HyGAnno on four datasets which are mouse brain, mouse lung, PBMC, and BMMC based on different parameter settings of graph embedding part. Both ACC and NMI are evaluated with the dimension number of the hidden layer d increasing from 64 to 256 and the learning rate l from* 10^{-5} *to* 10^{-3}.

Supplementary Tables

Supplementary Table 1: Properties of the constructed graphs

Datasets	Nodes (RNA graph)	Edges (RNA graph)	Nodes (ATAC graph)	Edges (ATAC graph)	Anchor cells (RNA)	Anchor cells (ATAC)	Connected edges
Mouse brain	8,055	50,028	8,055	103,727	7,617	7,316	23,106
Mouse lung	2,623	33,131	7,499	85,966	2,618	6,305	49,115
PBMC	13,345	164,947	7,828	112,848	10,911	6,949	23,725
BMMC	11,884	157,846	14,753	221,550	11,850	12,725	122,025
Breast cancer	15,088	150,044	11,116	131,879	14,175	9,324	84,520

Supplementary Table 2: Descriptions of datasets

Datasets	Types	Usages	Cells	Genes	Peaks	Cell types	Gene activity	Sources
Mouse brain (mm10)	scRNA-seq	Reference	8,055	33,160	N/A	14	N/A	Chen et al., 2019
Mouse lung (mm10)	scRNA-seq	Reference	2,623	23,433	N/A	8	N/A	Schaum et al., 2018
PBMC (hg19)	scRNA-seq	Reference	13,345	20,287	N/A	10	N/A	Granja et al., 2019
BMMC (hg19)	scRNA-seq	Reference	11,884	20,287	N/A	13	N/A	Granja et al., 2019
Breast cancer (hg19)	scRNA-seq	Reference	15,088	27,719	N/A	7	N/A	Wu et al., 2021
PBMC Rep1 (hg19)	scATAC-seq	Reference	9,060	N/A	127,541	6	Cicero	Satpathy et al., 2019
Mouse brain (mm10)	scATAC-seq	Target	8,055	N/A	267,670	14	Signac	Chen et al., 2019
Mouse lung (mm9)	scATAC-seq	Target	7,499	N/A	436,206	8	Cicero	Cusanovich et al., 2018
PBMC (hg19)	scATAC-seq	Target	7,828	N/A	452,004	10	Cicero	Granja et al., 2019
BMMC (hg19)	scATAC-seq	Target	14,753	N/A	452,004	13	Cicero	Granja et al., 2019
Breast cancer (hg19)	scATAC-seq	Target	11,116	N/A	155,403	7	Signac	Kumegawa et al., 2022
PBMC D10T1 (hg19)	scATAC-seq	Reference/ Target	2,588	N/A	452,004	9	Cicero	Granja et al., 2019
PBMC D12T1,2,3 (hg19)	scATAC-seq	Reference/ Target	3,070	N/A	452,004	9	Cicero	Granja et al., 2019

Supplementary Table 3: Prediction performance according to different numbers of anchor cells

		Mouse brain	Mouse lung	PBMC	BMMC
$k_2 = 5$	Anchor cells (RNA)	7,617	2,295	10,911	10,073
	Anchor cells (ATAC)	7,316	3,211	6,949	8,591
	Connected edges	23,106	6,937	23725	23,691
	ACC	0.833	0.737	0.921	0.821
	NMI	0.687	0.643	0.852	0.798
$k_2 = 10$	Anchor cells (RNA)	7,957	2,523	12,798	11,533
	Anchor cells (ATAC)	7,891	4,625	7,569	10,861
	Connected edges	50,683	16,363	52,326	54,206
	ACC	0.835	0.762	0.919	0.871
	NMI	0.661	0.652	0.849	0.818
$k_2 = 15$	Anchor cells (RNA)	8,008	2,586	13,190	11,779
	Anchor cells (ATAC)	7,997	5,398	7,705	11,991
	Connected edges	79,057	26,664	82,217	87,049
	ACC	0.837	0.767	0.928	0.873
	NMI	0.661	0.663	0.854	0.815
$k_2 = 20$	Anchor cells (RNA)	8,029	2,610	13,283	11,850
	Anchor cells (ATAC)	8,018	5,942	7,764	12,725
	Connected edges	108,061	37,675	112,835	122,025
	ACC	0.839	0.803	0.922	0.901
	NMI	0.665	0.701	0.840	0.823
$k_2 = 25$	Anchor cells (RNA)	8,037	2,618	13,324	11,877
	Anchor cells (ATAC)	8,034	6,305	7,789	13,191
	Connected edges	137,578	49,115	144,072	158,148
	ACC	0.834	0.823	0.915	0.865
	NMI	0.660	0.689	0.819	0.804

References

1. *Stuart, T., Srivastava, A., Madad, S., Lareau, C. A. & Satija, R. Single-cell chromatin state analysis with Signac. Nat. Methods* ***18****, 1333–1341 (2021).*
2. *Kipf, T. N. & Welling, M. Variational Graph Auto-Encoders. 1–3 (2016).*
3. *Kingma, D. P. & Welling, M. Auto-encoding variational bayes. 2nd Int. Conf. Learn. Represent. ICLR 2014 - Conf. Track Proc. 1–14 (2014).*
4. *Li, H. et al. Inferring transcription factor regulatory networks from single-cell ATAC-seq data based on graph neural networks. Nat. Mach. Intell.* ***4****, 389–400 (2022).*
5. *Chen, S., Lake, B. B. & Zhang, K. High-throughput sequencing of the transcriptome and chromatin accessibility in the same cell. Nat. Biotechnol.* ***37****, 1452–1457 (2019).*
6. *Schaum, N. et al. Single-cell transcriptomics of 20 mouse organs creates a Tabula Muris. Nature* ***562****, 367–372 (2018).*
7. *Cusanovich, D. A. et al. A Single-Cell Atlas of In Vivo Mammalian Chromatin Accessibility. Cell* ***174****, 1309-1324.e18 (2018).*
8. *Pliner, H. A. et al. Cicero Predicts cis-Regulatory DNA Interactions from Single-Cell Chromatin Accessibility Data. Mol. Cell* ***71****, 858-871.e8 (2018).*
9. *Granja, J. M. et al. Single-cell multiomic analysis identifies regulatory programs in mixed-phenotype acute leukemia. Nat. Biotechnol.* ***37****, 1458–1465 (2019).*
10. *Satpathy, A. T. et al. Massively parallel single-cell chromatin landscapes of human immune cell development and intratumoral T cell exhaustion. Nat. Biotechnol.* ***37****, 925–936 (2019).*
11. *Wu, S. Z. et al. A single-cell and spatially resolved atlas of human breast cancers. Nat. Genet.* ***53****, 1334–1347 (2021).*
12. *Nofech-Mozes, I., Soave, D., Awadalla, P. & Abelson, S. Pan-cancer classification of single cells in the tumour microenvironment. bioRxiv 2022.06.14.496107 (2022) doi:10.1038/s41467-023-37353-8.*
13. *Stuart, T. et al. Comprehensive Integration of Single-Cell Data. Cell* ***177****, 1888-1902.e21 (2019).*
14. *Lin, Y. et al. scJoint integrates atlas-scale single-cell RNA-seq and ATAC-seq data with transfer learning. Nat. Biotechnol.* ***40****, 703–710 (2022).*
15. *Song, Q., Su, J. & Zhang, W. scGCN is a graph convolutional networks algorithm for knowledge transfer in single cell omics. Nat. Commun.* ***12****, 1–11 (2021).*
16. *Rousseeuw, P. J. Silhouettes: A graphical aid to the interpretation and validation of cluster analysis. J. Comput. Appl. Math.* ***20****, 53–65 (1987).*
17. *Schep, A. N., Wu, B., Buenrostro, J. D. & Greenleaf, W. J. ChromVAR: Inferring transcription-factor-associated accessibility from single-cell epigenomic data. Nat. Methods* ***14****, 975–978 (2017).*
18. *Haghverdi, L., Büttner, M., Wolf, F. A., Buettner, F. & Theis, F. J. Diffusion pseudotime robustly reconstructs lineage branching. Nat. Methods* ***13****, 845–848 (2016).*
19. *Kumegawa, K. et al. GRHL2 motif is associated with intratumor heterogeneity of cis-regulatory elements in luminal breast cancer. npj Breast Cancer* ***8****, (2022).*
